# Transcriptomic heterogeneity of non-beta islet cells is associated with type 2 diabetes development in mouse models

**DOI:** 10.1007/s00125-024-06301-6

**Published:** 2024-11-07

**Authors:** Pascal Gottmann, Thilo Speckmann, Mandy Stadion, Prateek Chawla, Judith Saurenbach, Nikolay Ninov, Heiko Lickert, Annette Schürmann

**Affiliations:** 1 Department of Experimental Diabetology, German Institute of Human Nutrition Potsdam-Rehbruecke (DIfE), Nuthetal, Germany; 2 German Center for Diabetes Research (DZD), München Neuherberg, Germany; 3 Center for Regenerative Therapies TU Dresden, Dresden, Germany; 4 Institute of Diabetes and Regeneration Research, Helmholtz Center Munich, Neuherberg, Germany; 5 Institute of Stem Cell Research, Helmholtz Center Munich, Neuherberg, Germany; 6 University of Potsdam, Institute of Nutritional Sciences, Nuthetal, Germany

**Keywords:** Beta cell protection, Cell–cell communication, Heterogeneity in islets, Macrophages, Type 2 diabetes

## Abstract

**Aims/hypothesis:**

The aim of this work was to understand the role of non-beta cells in pancreatic islets at early stages of type 2 diabetes pathogenesis.

**Methods:**

Specific clustering was employed to single-cell transcriptome data from islet cells of obese mouse strains differing in their diabetes susceptibility (diabetes-resistant B6.V.*Lep*^ob/ob^ [OB] and diabetes-susceptible New Zealand Obese [NZO] mice) on a diabetogenic diet.

**Results:**

Refined clustering analysis revealed several heterogeneous subpopulations for alpha cells, delta cells and macrophages, of which 133 mapped to human diabetes genes identified by genome-wide association studies. Importantly, a similar non-beta cell heterogeneity was found in a dataset of human islets from donors at different stages of type 2 diabetes. The predominant alpha cell cluster in NZO mice displayed signs of cellular stress and lower mitochondrial capacity (97 differentially expressed genes [DEGs]), whereas delta cells from these mice exhibited higher expression levels of maturation marker genes (*Hhex* and *Sst*) but lower somatostatin secretion than OB mice (184 DEGs). Furthermore, a cluster of macrophages was almost twice as abundant in islets of OB mice, and displayed extensive cell–cell communication with beta cells of OB mice. Treatment of beta cells with IL-15, predicted to be released by macrophages, activated signal transducer and activator of transcription (STAT3), which may mediate anti-apoptotic effects. Similar to mice, humans without diabetes possess a greater number of macrophages than those with prediabetes (39 mmol/mol [5.7%] < HbA_1c_ < 46 mmol/mol [6.4%]) and diabetes.

**Conclusions/interpretation:**

Our study indicates that the transcriptional heterogeneity of non-beta cells has an impact on intra-islet crosstalk and participates in beta cell (dys)function.

**Data availability:**

scRNA-seq data from the previous study are available in gene expression omnibus under gene accession number GSE159211 (https://www.ncbi.nlm.nih.gov/geo/query/acc.cgi?acc=GSE159211).

**Graphical Abstract:**

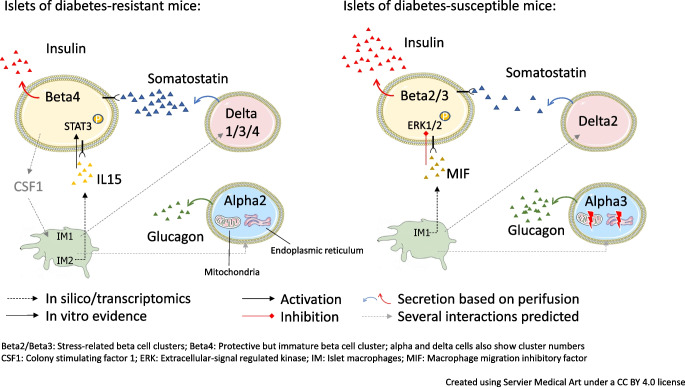

**Supplementary Information:**

The online version contains peer-reviewed but unedited supplementary material available at 10.1007/s00125-024-06301-6.


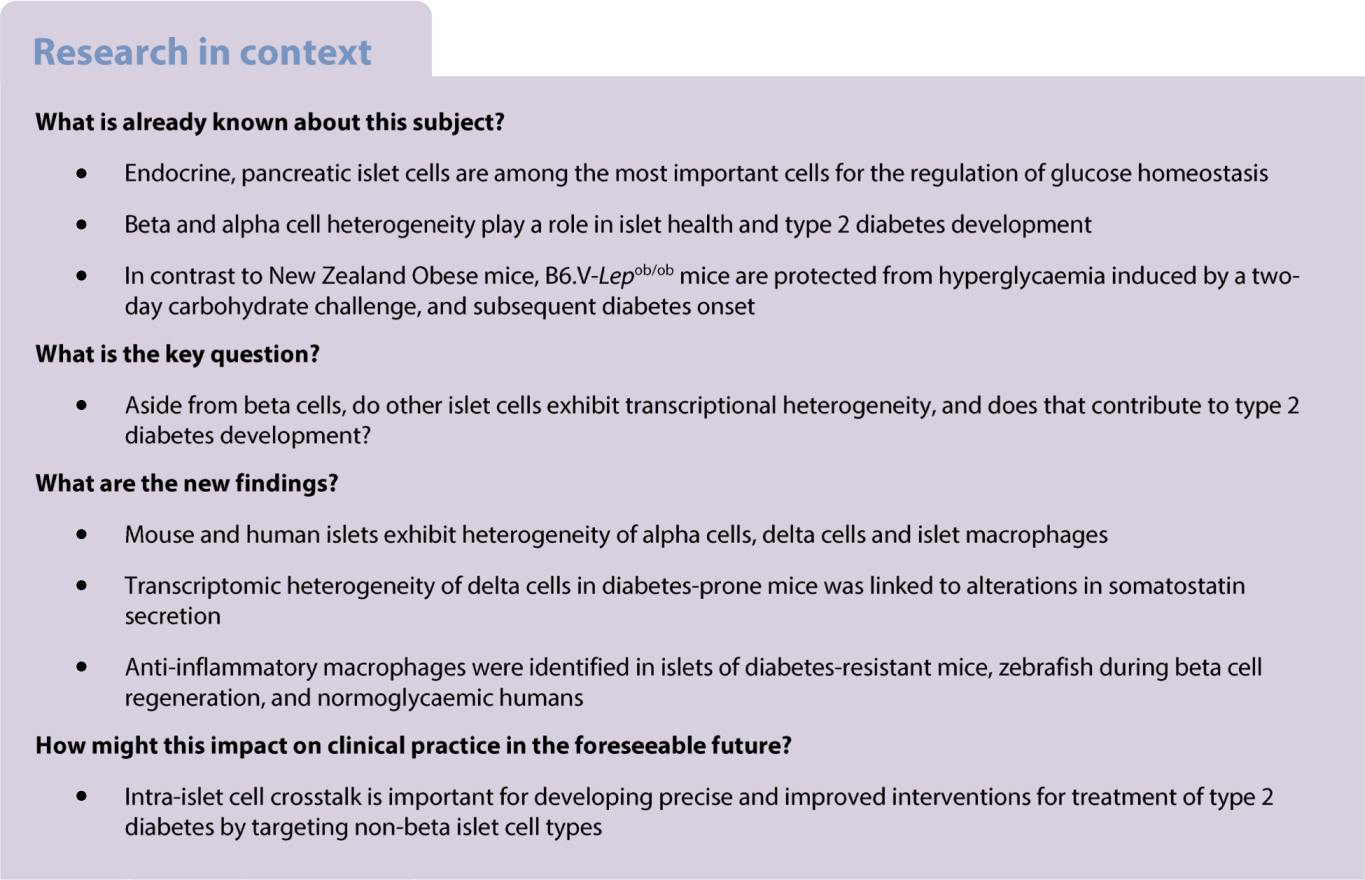


## Introduction

Diabetes is one of the four major non-communicable diseases. In 2019, it was estimated to be a direct cause of death for 1.6 million people worldwide [1]. This number rises even further if including deaths due to diabetes-associated complications, which can also have a severe impact on the quality of life of those affected. Type 2 diabetes is the most common subtype of this metabolic disease, which itself can be stratified into four clusters [2]. Overall, type 2 diabetes presents with a heterogenous range of phenotypes and contributions from genetic and environmental factors. A major cause and driver of disease progression, in particular for the severe insulin-deficient diabetes (SIDD) cluster, is the dysfunction of endocrine pancreatic islet cells, which play a major role in regulating glucose homeostasis.

Single-cell RNA sequencing (scRNA-seq) is a powerful technology that helps to elucidate the impact of different cell types on disease development and the study of tissue heterogeneity at the single-cell level. This is of particular interest for islets, which contain mixed endocrine cell populations involved in regulation of blood glucose concentration [3]. However, due to the genetic and phenotypic heterogeneity of type 2 diabetes in humans, scRNA-seq of islets from humans with and without type 2 diabetes revealed only a marginal overlap of differentially expressed genes (DEGs) [4, 5]. To better understand human diabetes pathogenesis, we utilised two genetically discrete, obese mouse models with differences in diabetes susceptibility. In a previous study, we showed that islets from diabetes-resistant B6.V.*Lep*^ob/ob^ (OB) and diabetes-susceptible New Zealand Obese (NZO) mice share features with human type 2 diabetes under a synchronised, 2 day glucotoxic stimulus [6]. Furthermore, several of our mouse studies identified diabetes genes with potential translational evidence for processes including beta cell proliferation (*Lefty1*, *Apoa2*, *Pcp4l1*) [7], cilia function (*Kif3a*) [8] and beta cell survival and insulin secretion (*Gjb4*) [9].

Pancreatic islet macrophages play a crucial role in maintaining islet cell homeostasis, immune surveillance and the modulation of inflammatory responses [10]. While a lack of macrophages may be beneficial for reducing autoimmune diabetes incidence in NOD mice, a model of type 1 diabetes [11], certain levels of inflammation are beneficial for glucose metabolism by preserving beta cell mass and function. In recent years, this balance between constructive and destructive islet inflammation has become a focal point in diabetes research, which has increasingly recognised an inflammatory component of type 2 diabetes [12, 13]. As the principal resident immune-cell population, islet macrophages have been shown to play an important role in maintaining islet homeostasis. In this context, both their number and polarisation state determine the balance between constructive [14–20] and destructive inflammation, especially driven by obesity [13, 21–23], and its subsequent impact on insulin secretion, beta cell differentiation, proliferation and survival. Further characterising their molecular signature and transcriptional response to obesity and diabetogenic stimuli is important in understanding their impact on type 2 diabetes pathogenesis.

Performing scRNA-seq of islets from those obese mouse strains, we previously were able to identify four highly abundant, discrete beta cell clusters based on diet and genotype [24]. Beta cells of both mouse strains on non-diabetogenic carbohydrate-free high-fat diet (−CH) shared a common beta cell cluster (Beta1). In contrast, feeding a carbohydrate-containing diabetogenic diet (+CH) resulted in the emergence of a protective but immature beta cell cluster in OB mice (Beta4), whereas beta cells of NZO were assigned to two stress-related clusters (Beta2 and Beta3). Finally, this beta cell heterogeneity was associated with human type 2 diabetes. Among other studies [25, 26], this work raises the question of whether non-beta cells exhibit similar heterogeneity and how such cell-type-specific heterogeneity might be relevant to type 2 diabetes pathogenesis.

## Methods

### Animals

Animals used for this study included male NZO/HIBomDife (R. Kluge, German Institute of Human Nutrition Potsdam-Rehbrücke, Germany [27]) and C57BL/6J (B6; Charles River Laboratories, Germany, strain code 632; https://www.criver.com/products-services/find-model/jax-c57bl6j-mice?region=23) mice from our own breeding colonies, as well as B6.V-*Lep*^ob/ob^ mice (Charles River Laboratories, Italy, strain code 606; https://www.criver.com/products-services/find-model/jax-obob-mice?region=23). B6 mice were kept on a standard diet, whereas NZO and OB mice were on a dietary intervention, as described previously [24]. Animals were kept in groups of two to five in individually ventilated cages (IVCs) with ad libitum access to food and water. Cages were housed in a climate-controlled facility (22±2°C, 40–50% humidity) with a 12 h light–dark cycle. All animal care was in accordance with the guidelines of the state of Brandenburg, Germany (approval number 2347-33-2019).

#### scRNA-seq analysis of mouse islet cells

We used scRNA-seq data from our previous study [24]. Quality control steps, including filtering, normalisation, batch correction and cell-type identification, were not changed [24, 28]. Instead, to gain more insight into islet cell heterogeneity, alpha cells, delta cells and pancreatic polypeptide cells as well as macrophages were clustered in independent steps using a higher resolution than before. Afterwards, DEGs between the clusters of different islet cell types were estimated using the MAST R-package as described previously [24].

#### scRNA-seq analysis of human islet cells

Single-cell data were obtained from the Gene Expression Omnibus (GEO; www.ncbi.nlm.nih.gov/geo/) under accession ID GSE200044 in FASTQ format [29]. The sequencing reads were aligned to the human genome build GRCh38 using Cell Ranger version 7.1 (10x Genomics, USA). Subsequent data analysis was conducted following the same procedures as described for the mouse islets. Prediabetes was defined according to Wang et al [29] via no prior type 2 diabetes diagnosis and HbA_1c_ levels between 39 mmol/mol [5.7%] and 46 mmol/mol [6.4%].

#### scRNA-seq analysis of zebrafish islet cells

scRNA-seq data from GEO datasets GSM5060846 and GSM5060847 were downloaded and only time points 0 and 2 days post ablation (dpa) were analysed by Scanpy (v1.9.1) [28]. Quality control was done as described by Singh et al [30], reproducing their results. A second round of clustering similar to that described for the mouse data was only applied to the immune-cell cluster.

### Interpretation of scRNA-seq expression data

Overall expression data received from scRNA-seq can be interpreted as unitless, as normalisation methods can vary and may involve different strategies such as scaling, log transformation or adjusting for sequencing depth. Therefore, the term ‘normalised read counts’ lacks a standardised definition, and its interpretation may differ depending on the specific normalisation technique applied in the data processing pipeline. This is reflected in our labelling by using terms ‘scaled mean expression’ (if normalised read count are scaled per gene) and ‘mean expression’ (normalised read counts).

#### Estimating cell–cell communication

Cell–cell communication between the different macrophage clusters and other islet cell clusters was assessed based on known ligand–receptor interactions using CellPhoneDB version 2 [31] with default settings. Further, NicheNet version 2.0.4 [32] was used to identify targets located downstream of ligands.

#### Marker gene detection for clusters

To identify marker genes for specific cell-type subpopulations, only cells belonging to the respective cell type were considered. Afterwards rank_genes_groups of the Scanpy package was used via ‘*t* test-overestim_var’. Genes were filtered for the top ten markers, and only those were visualised using the Scanpy dotplot function.

#### Comparison with human type 2 diabetes risk genes

DEGs of specific cell types were compared with human type 2 diabetes risk genes from a prominent meta study [9] and the NHGRI-EBI GWAS catalogue [10].

#### Cell culture

Mycoplasma-free MIN6 cells (AddexBio, USA; C0018008) were cultured in Advanced Medium (AddexBio C0003–04) containing 15% (vol./vol.) heat-inactivated FCS (Gibco, USA; 10270106) and 0.05 mmol/l 2-mercaptoethanol (Gibco 21985023) in a humidified incubator at 37°C and 5% CO_2_.

#### Islet isolation and dispersion

Islet isolation diet was performed via injection of collagenase (Roche, Switzerland; 11249002001) solution (0.3 mg/ml) into the common bile duct, as described [7]. For dispersion, islets of 17- to 23-week-old male B6 mice on standard diet were incubated in Accutase solution (PAN Biotech, Germany; P10–21100; 75 µl per 100 islets) using gentle agitation in a water bath at 37°C for up to 15 min. The reaction was stopped by adding pre-warmed islet culture medium (RPMI1640 [PAN Biotech P04–16500], 10% [vol./vol.] FCS Gold [PAA Laboratories, Germany; A15–151], 100 U/ml penicillin + 0.1 mg/ml streptomycin [PAN Biotech P06–07100]). Total cell number was counted and islet cells were pelleted by centrifugation at 300 *g* for 5 min. The supernatant fraction was removed, cells were resuspended in culture medium (30 µl per 1.5×10^5^ cells) and then dispensed in a small volume onto extracellular matrix (ECM)/poly-l-lysine (PLL)-coated 24-well plates. After incubation for 2 h at 37°C and 5% CO_2_ for 2 h, culture medium was topped up and cells were left to recover overnight.

#### Islet perifusion assay

The perifusion experiment [8, 33, 34] was performed on 30 freshly isolated islets from 18-week-old male OB and NZO mice fed with either −CH or +CH (Altromin, Germany). Following a 30 min recovery period in islet culture medium, islets were equilibrated in KRBH with 2.8 mmol/l glucose (KRBH-2.8) at 37°C for 30 min. Subsequently, 30 islets were loaded into a perifusion chamber (PERI5; Biorep Technologies, USA) with polyacrylamide bead solution (Bio-Gel P-4 Gel; Bio-Rad Laboratories, USA) and continuously perifused with KRBH-2.8 at a flow rate of 100 μl/min for 60 min. Following this, flow-through fractions were collected at 3 min intervals, with KRBH containing the following: (1) 2.8 mmol/l glucose for 15 min; (2) 20 mmol/l glucose for 30 min; (3) 20 mmol/l glucose and 100 nmol/l somatostatin (Bachem, Switzerland) for 30 min; and (4) 2.8 mmol/l glucose for 15 min. Due to the dead volume of the machine, the delay of stimulation media reaching the perifusion chamber was about 5 min. Finally, islets were retrieved in 500 µl acid ethanol (0.18 mol/l HCl in 70% [vol./vol.] EtOH).

#### Measurement of insulin, glucagon and somatostatin

Insulin levels in mouse plasma and islet perifusion fractions were measured using the Ultrasensitive Insulin ELISA kit (Alpco Diagnostics, USA) and normalised to residual insulin content. Glucagon (Mercodia, Sweden) and somatostatin (Phoenix Pharmaceuticals, USA) levels in OB and NZO mouse plasma and islet perifusion fractions were measured via ELISA according to manufacturer instructions.

#### Ligand treatment and western blotting

MIN6 cells or dispersed B6 islets were pre-incubated in serum-free Advanced Medium or RPMI 1640 (PAN Biotech no. P04–16500) for 6 h, followed by treatment with indicated amounts of MIF (macrophage migration inhibitory factor) or IL-15 (Bio-Techne, USA; 1978-MF-025/CF or 447-ML-010/CF) in serum-free medium for 20 min. Fluorescence signals were detected using an Odyssey CLx imager and quantified using Image Studio (LI-COR Biosciences, USA). Immunoblot was performed as described previously [35], using primary antibodies against phospho-STAT3 (signal transducer and activator of transcription 3) (Cell Signaling Technology, USA; 9131, 1:200), total STAT3 (Cell Signaling Technology 9132, 1:1000), phospho-ERK1/2 (Cell Signaling Technology 4377, 1:1000) and total ERK1/2 (Cell Signaling Technology 4696, 1:2000). Secondary antibodies were conjugated to fluorescent probes (anti-mouse, Thermo Fisher Scientific, USA; 35519 or SA535521; anti-rabbit, Thermo Fisher Scientific 35568 or SA5–35571; all 1:10,000). Phospho-specific primary antibodies were diluted in 5% (wt/vol.) BSA (PAN Biotech P06-1391500) in TBS-T (10 mmol/l Tris, 150 mmol/l NaCl, 0.1% [vol./vol.] Tween-20); all other primary and secondary antibodies were diluted in 5% (wt/vol.) skim milk powder (Carl Roth, Germany; T145.2) in TBS-T.

#### Islet immunostaining

Pancreases from 18-week-old male OB and NZO ±CH mice were harvested, fixed and immunostaining performed as described previously [7] on pancreatic sections (4 µm thick). Images were acquired using a confocal microscope (TCS SP8 X; Leica Microsystems, Germany). Primary antibodies were directed against somatostatin (Invitrogen, USA; MA5–16987, 1:500), secretogranin V (SCG5, Proteintech, USA; 10761–1-AP, 1:100), tetraspanin 8 (TSPAN8; Invitrogen PA5–75311, 1:100), insulin (Sigma-Aldrich, USA; I2018, 1:10,000) or F4/80 (AbD Serotec, USA; MCA497, 1:50). Primary antibodies were detected with fluorophore-conjugated secondary antibodies, at a dilution of 1:400 (Alexa Fluor 488, Alexa Fluor 546 and Alexa Fluor 647; Invitrogen), plus DAPI (1 μg/ml).

### Plotting

Ligands and their putative downstream genes were visualised by drawing a heatmap using the ‘make_heatmap_ggplot’ function of the NicheNet R-package version 2.0.4 [32]. Ligand–receptor interactions detected via CellPhoneDB were plotted using R version 4.2.2 with the circlize-package version 0.4.15. Otherwise, plots were generated using R version 4.2.2. Bar plots of cell-type composition were drawn using the barplot function of R after reading in the Python adata object with the rhdf5-package version 2.34.0. Pie-charts were plotted using the R pie function. Dotplots were drawn using the methods integrated in Scanpy or the R-package of ggplot2 version 3.4.0.

#### Pathway enrichment analysis

Pathway enrichment analysis for Kyoto Encyclopedia of Genes and Genomes (KEGG) terms was performed using the database for annotation visualisation and integrated discovery (DAVID) in version v2022q3.3.

## Results

### Clustering of less-frequent islet cells in diabetes-resistant and diabetes-susceptible mice

**Fig. 1:**
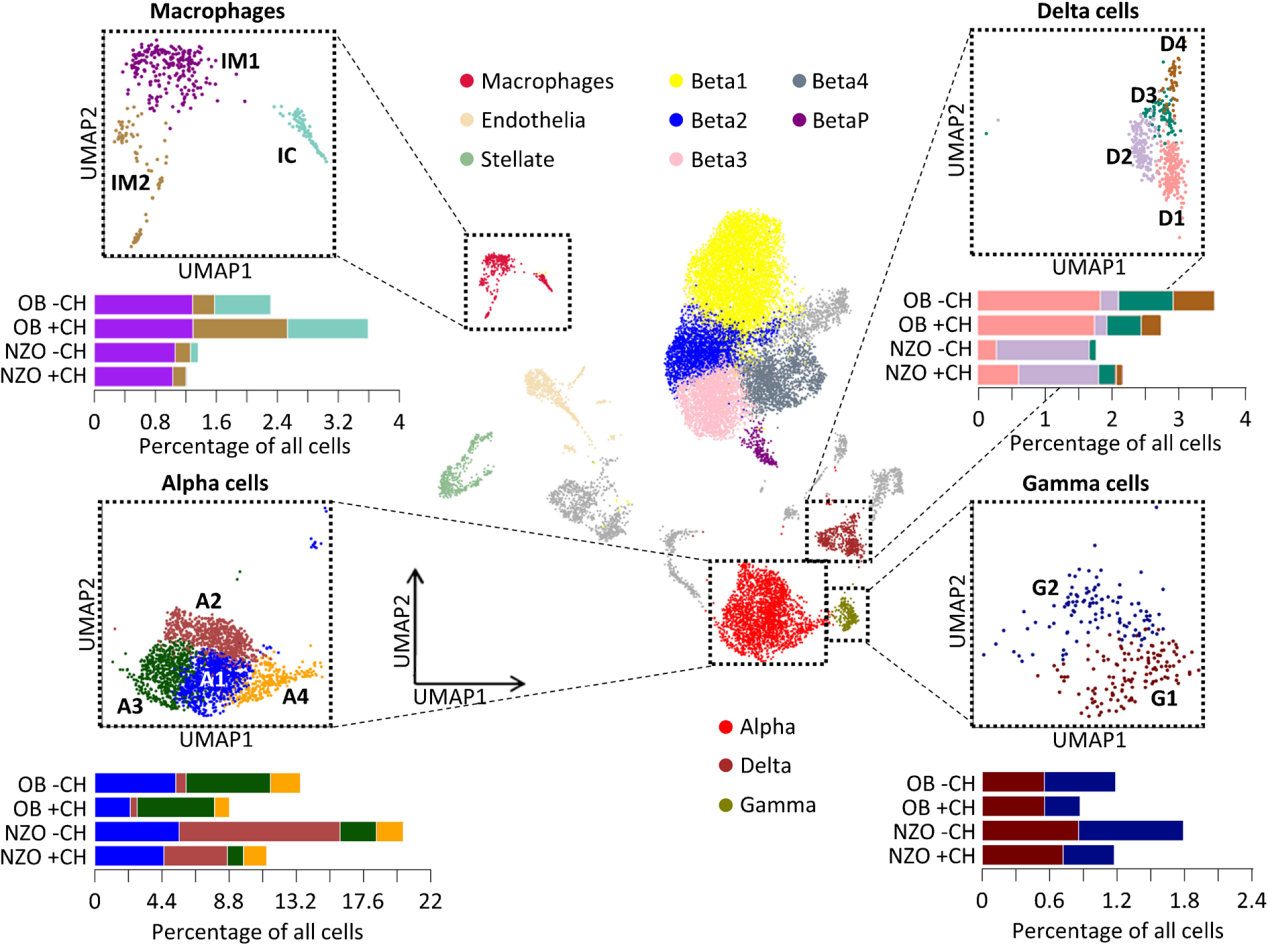
Refined clustering and cell composition of islets from NZO and OB mice fed with and without diabetogenic diet. Clusters of islet macrophages (IM1–2), immune cells (IC), delta cells (D1–4), alpha cells (A1–4) and gamma cells (G1–2) are shown. Original Louvain clustering is located in the centre; refined clusters for the respective cell types are depicted in dotted boxes. UMAP, uniform manifold approximation and projection

In a previous study, we mainly focused on beta cell transcriptomics and identified several diet- and genotype-specific clusters [24]. Here, we aimed to clarify the impact of less-frequent islet endocrine and immune-cell populations (alpha cells, delta cells, gamma cells and macrophages; electronic supplementary material [ESM] Fig. 1) on type 2 diabetes development. Therefore, Louvain clustering was applied a second time to verify that under different feeding conditions (−CH, +CH) these non-beta islet cells also form different clusters. Four clusters (A1–A4) were detected among glucagon-secreting alpha cells, all of which were reduced under +CH feeding in both mouse strains (especially A1 in OB and A2 in NZO mouse islets). Diabetes-susceptible NZO mice showed a high abundance of alpha cells assigned to the A2 cluster, whereas islets of diabetes-resistant OB mice had more A3 cells (Fig. 1). Notably, somatostatin-secreting delta cells, for which we detected four clusters (D1–D4), were more abundant in OB mice (based on transcriptome). The major delta cell clusters in OB mouse islets were D1, D3 and D4, whereas in NZO mouse islets, D2 was most prevalent (Fig. 1). There were no clear differences between OB and NZO mouse gamma cells, since both strains showed a decrease in G2 on the diabetogenic diet (+CH; Fig. 1). Finally, transcriptomics indicated a higher number of cells belonging to islet macrophage clusters (islet macrophage cluster 1 [IM1] and islet macrophage cluster 2 [IM2]) and an immune-cell cluster in islets of OB mice compared with NZO mice; IM2 cells in particular were increased in OB mice fed with +CH (Fig. 1). Similar subpopulations were identified by applying Louvain clustering to each mouse model on the respective diet (not shown). Overall, this analysis revealed differences in alpha cells, delta cells and macrophage clusters. Most differences were already apparent with −CH feeding, indicating a strong genetic component. At least for macrophages, the cluster composition differences between OB and NZO mice were further exacerbated by diabetogenic diet.

### Genetic predisposition and heterogeneity of human non-beta islet cells

**Fig. 2:**
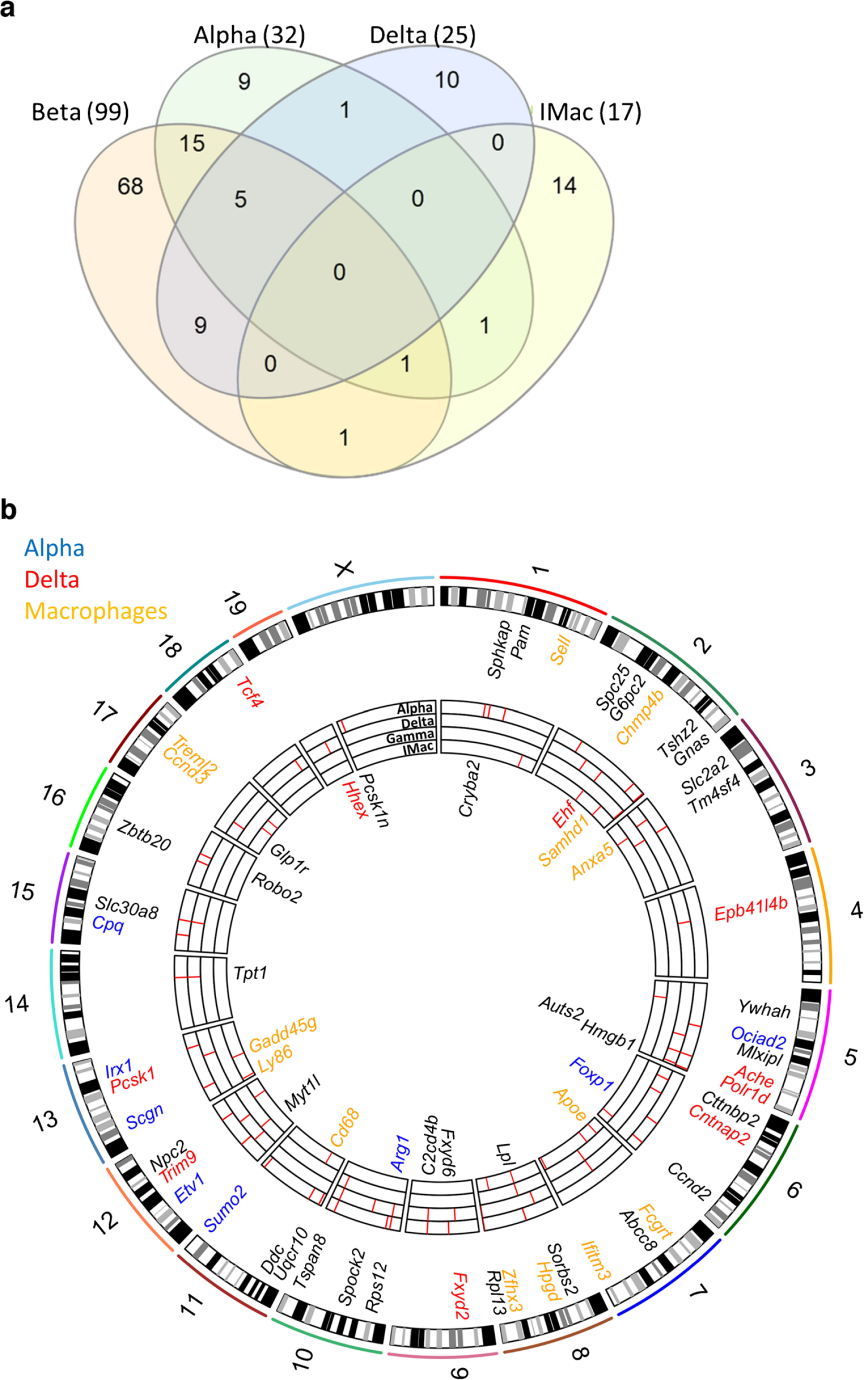
Human type 2 diabetes GWAS genes mapped to specific cell types. (**a**) Venn diagram illustrating DEGs identified in cell-type-specific clusters compared with known human type 2 diabetes risk genes. Orange, green, blue and yellow represent beta cells, alpha cells, delta cells and macrophages (iMac), respectively. (**b**) Circos plot depicting mouse DEGs associated with human type 2 diabetes in specific cell types. The plot indicates different cell types from outside to inside as follows: alpha cells, delta cells, gamma cells and macrophages

Next, genes differentially expressed between specific NZO and OB mouse cell types were compared with known human type 2 diabetes genome-wide association study (GWAS) genes from the NHGRI-EBI GWAS catalogue [36] and a recently published large-scale type 2 diabetes meta-analysis [37]. A total of 133 genes associated with human type 2 diabetes were differentially expressed in at least one islet cell type (99 in beta cells, 32 in alpha cells, 25 in delta cells, five in gamma cells (data not shown) and 17 in macrophages) of OB and NZO mice (Fig. 2a). With a focus on cell-type-specific type 2 diabetes risk genes, we identified 32 genes (Fig. 2b): seven for alpha cells (*Arg1*, *Cpq*, *Etv1*, *Foxp1*, *Ociad2*, *Scgn* and *Sumo2*); ten for delta cells (*Ache*, *Cntnap2, Ehf, Epb41l4b*, *Fxyd2*, *Hhex*, *Pcsk1*, *Polr1d, Tcf4* and *Trim9*); none for gamma cells; and 14 for macrophages (*Anxa5*, *Apoe*, *Ccnd3*, *Cd68*, *Chmp4b*, *Fcgrt*, *Gadd45g*, *Hpgd*, *Ifitm3*, *Ly86*, *Samhd1*, *Sell*, *Treml2*, *Zfhx3*). *Hhex* is a well-known delta cell-specific diabetes gene [38], providing a proof of concept. In addition, we discovered seven other potential key transcriptional regulators of gene expression networks in type 2 diabetes (ESM Fig. 2): *Etv1*, *Irx1* and *Foxp1* (alpha cells); *Ehf*, *Hhex* and *Tcf4* (delta cells); and *Zfhx3* (macrophages).

**Fig. 3:**
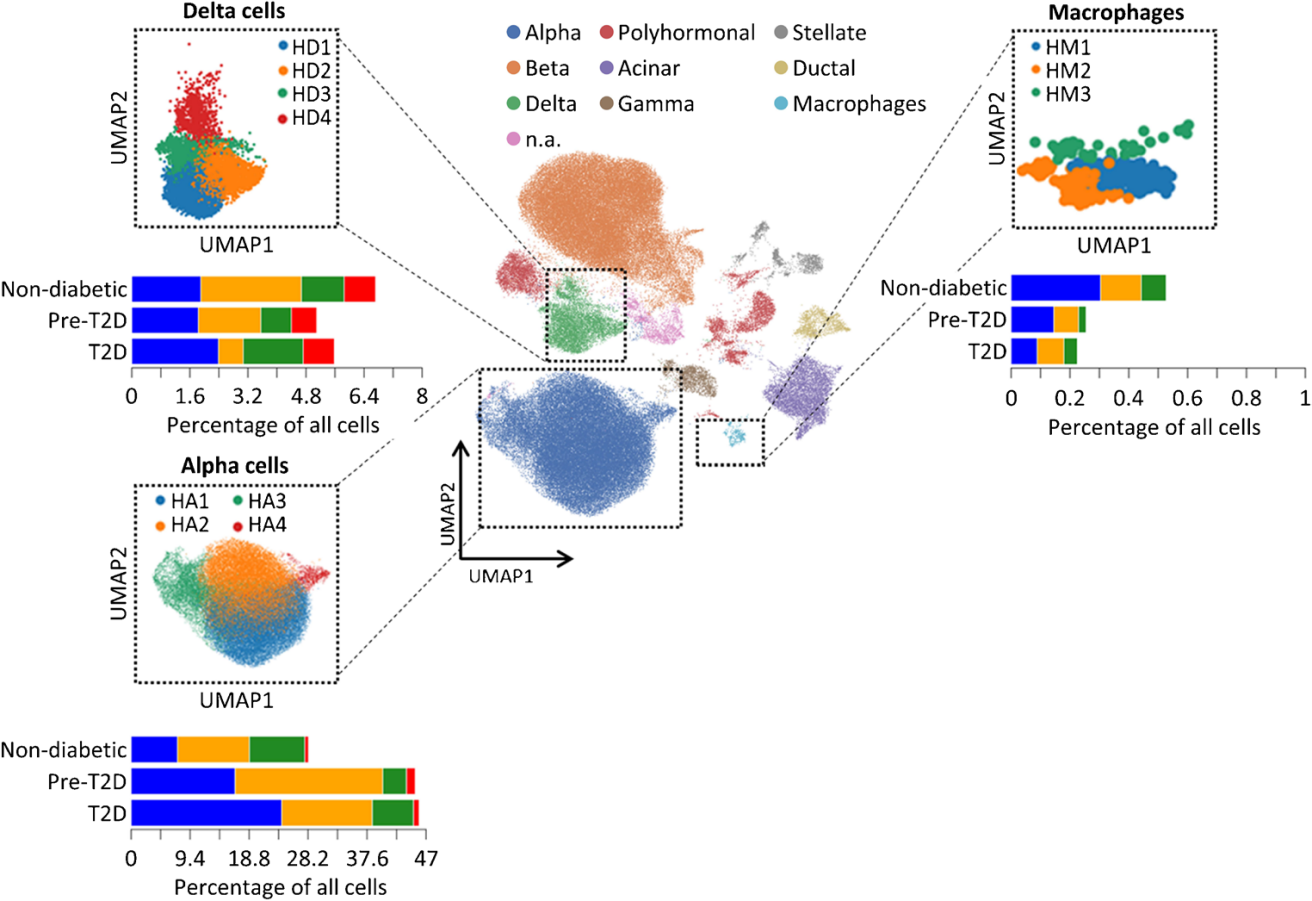
Refined clustering and cell composition of islets from control donors and donors with prediabetes or diabetes. Human macrophages (HM1–3), delta cell clusters (HD1–4) and alpha cell clusters (HA1–4) are shown. Original Louvain clustering is located in the centre, and refined clusters for the respective cell types are depicted in dotted boxes. n.a., not assigned; pre-T2D, prediabetes; T2D, type 2 diabetes; UMAP, uniform manifold approximation and projection

Overall, our data indicate that islet cell heterogeneity is an important aspect in the pathogenesis of type 2 diabetes, not only with respect to beta cells but also for alpha cells, delta cells and macrophages. We therefore sought to determine whether similar heterogeneity exists for these cells in human scRNA-seq. Thus, we used publicly available scRNA-seq data for pancreatic islets from control individuals and individuals with prediabetes (39 mmol/mol [5.7%] < HbA_1c_ < 46 mmol/mol [6.4%]) and diabetes, as described by Wang et al [29] (GSE200044, https://www.ncbi.nlm.nih.gov/geo). We performed Louvain clustering using the listed marker genes for defining the specific cell types (Fig. 3 and ESM Fig. 3a). In islets of people with prediabetes and type 2 diabetes, transcriptomics indicated a greater number of alpha cells overall, in particular those of cluster HA1. Concerning delta cells, islets of people with type 2 diabetes seem to exhibit a greater number of HD1 cells and a lower number of HD2 cells. Consistent with our observations in mouse models, the human islet transcriptome suggests a decrease in macrophages during disease progression. Thus, heterogeneity also exists in human non-beta cells.

### Divergent transcriptional adaptations of NZO and OB mouse alpha cells in response to diabetogenic diet

**Fig. 4:**
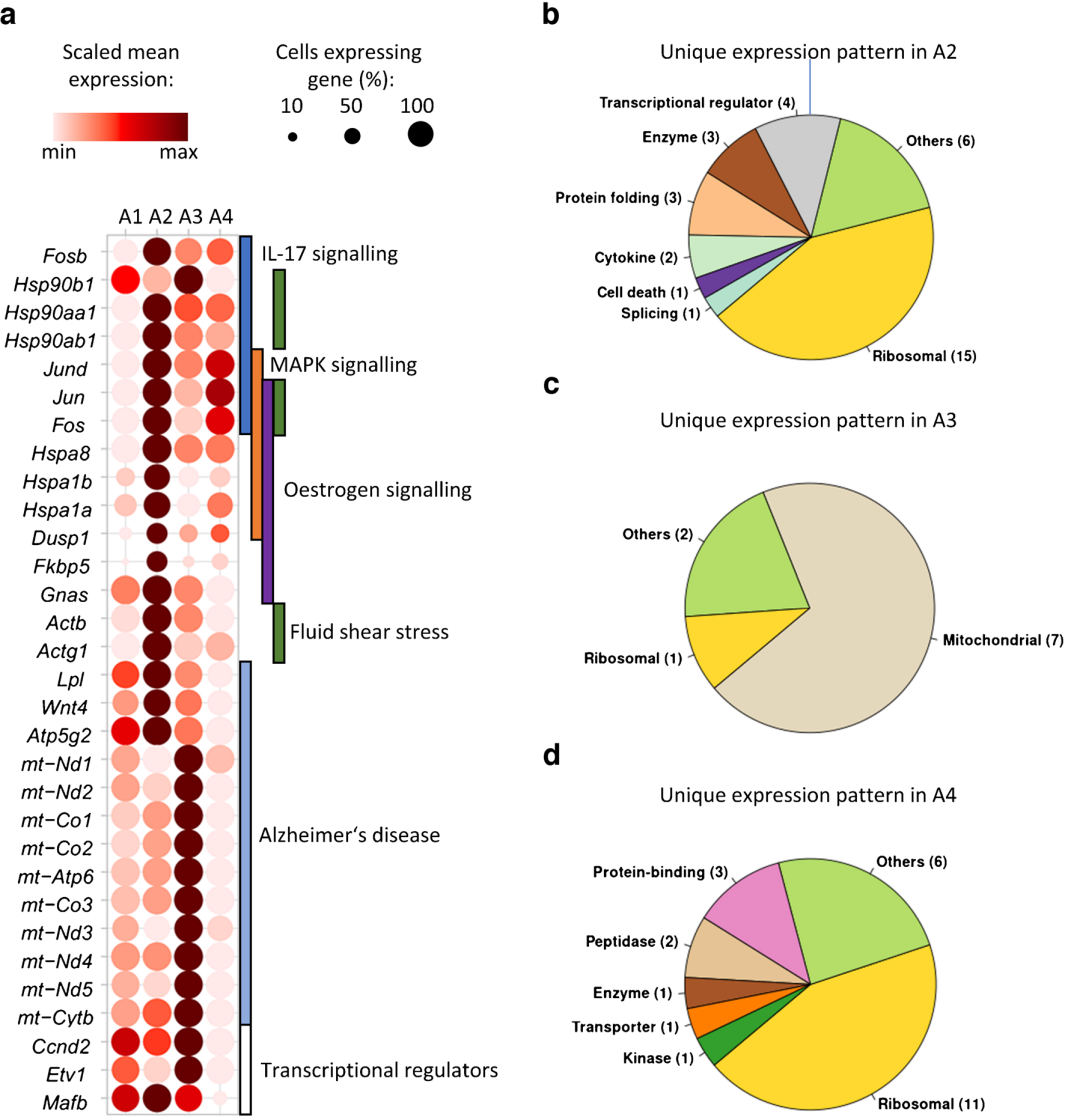
Pathway enrichment analysis of genes differentially expressed between alpha cell clusters A2 and A3 and classification of cluster-specific genes (A2–A4). (**a**) Dot plot of genes enriched in several type 2 diabetes-relevant pathways. Dot size reflects the number of cells expressing the indicated genes, while colour code depicts the mean expression levels within a cluster. (**b**, **c**, **d**) Pie-charts reflecting the functional annotation of genes with unique expression patterns for clusters A2 (**b**), A3 (**c**) and A4 (**d**)

Pancreatic alpha cell dysfunction is part of type 2 diabetes pathogenesis [39]. Thus, we studied the transcriptional response of alpha cells to diabetogenic diet in the two obese mouse strains, which differ in their diabetes susceptibility. Between the four alpha cell clusters, 250 genes were differentially expressed, corresponding to 40 different pathways (ESM Fig. 4a, ESM Table 1). Similar to observations made for beta cell clusters, ‘ribosome’ *(Rps27a*, *Rps7* and *Rpl7*) and ‘protein processing in endoplasmic reticulum’ (*Ssr4*, *Calr* and *Dnajb1*) were among the affected pathways, pointing to compensatory alterations in the translational machinery. Although our single-cell data indicated a decrease in alpha cells in mice of both strains fed with +CH, the expression pattern revealed marked differences in the clusters A2 (NZO-enriched) and A3 (OB-enriched) (Fig. 1). Of 97 DEGs between the two clusters, 13 were associated with mitochondrial function (e.g. *mt-Nd1–5*, *mt-Co1–3, mt-Atp6*) and others coded for heat shock proteins (*Hsp90b1*, *Hspa8*, *Hspa1a/b*) (Fig. 4a). The A2 cluster (NZO mice) displayed a higher percentage of cells expressing genes encoding heat shock protein family members (annotated to IL-17, mitogen-activated protein kinase [MAPK] and oestrogen signalling) (Fig. 4a) and AP-1 subunits (*Jun*, *Jund*, *Fos* and *Fosb*) than A3, therefore implicating this cluster in the regulation of proliferation, differentiation, endoplasmic reticulum stress and apoptosis [25, 40]. In contrast, mitochondrial genes associated with Alzheimer’s disease, oxidative phosphorylation and Parkinson’s disease were more abundant in the A3 cluster (OB mice), suggesting an adaptive lowering of glucagon secretion in OB mouse alpha cells through increased oxidative phosphorylation [41]. While plasma glucagon was generally lower in NZO than in OB mice, +CH feeding decreased glucagon levels in the OB mice while raising plasma glucagon in NZO mice, indicating that alpha cells of OB mice are still sensitive to suppression by glucose and insulin while alpha cells in NZO mice show signs of dysfunctional stimulus–secretion coupling (ESM Fig. 4b, c). In addition, several transcriptional regulators associated with cell cycle regulation (*Ccnd2*) or alpha cell maturity (*Etv1*, *Mafb*) were expressed at different levels among the different alpha cell clusters (Fig. 4a).

### Different expression patterns of the alpha cell clusters

To define the alpha cell clusters more precisely, we searched for unique expression patterns in A1–A4. Cluster A1 did not exhibit genes with a unique expression pattern compared with the other alpha cell clusters, indicating a basic cell population not altered on diabetogenic diet. For cluster A2, the most abundant alpha cell cluster in NZO, unique expression of genes included those encoding for several transcriptional regulators (*Fosb*, *Rbm39*, *Sox4*, *Zbtb20*) and chaperones (*Fkbp5*, *Hspa1b*, *Hspa8*), and those involved in secretion and signalling (*Scg2*, *Wnt4*) (Fig. 4b and ESM Table 1). These specific expression patterns may contribute to defects in stress response, protein folding and alpha cell maturity [25, 40]. They are in line with the lower expression levels of ribosomal genes (ESM Table 1) and lower overall number of genes expressed in A2 cells (ESM Fig. 4d). In the OB-enriched A3 cluster, most uniquely expressed genes were mitochondria-related, suggesting specific adaptation of OB mouse alpha cells to metabolic challenge via improved mitochondrial function (Fig. 4c). Finally, cluster A4 (similar abundance in both mouse strains) showed a unique expression pattern of 25 genes, including lower levels of mature alpha cell markers *Mafb* and *Gcg*, suggesting that these cells may be in a more transitional state (Fig. 4d). Altogether, these analyses revealed that stress responses and mitochondrial genes differed between alpha cells of OB and NZO mice.

### NZO mouse delta cells show altered expression of genes implicated in somatostatin secretion

**Fig. 5:**
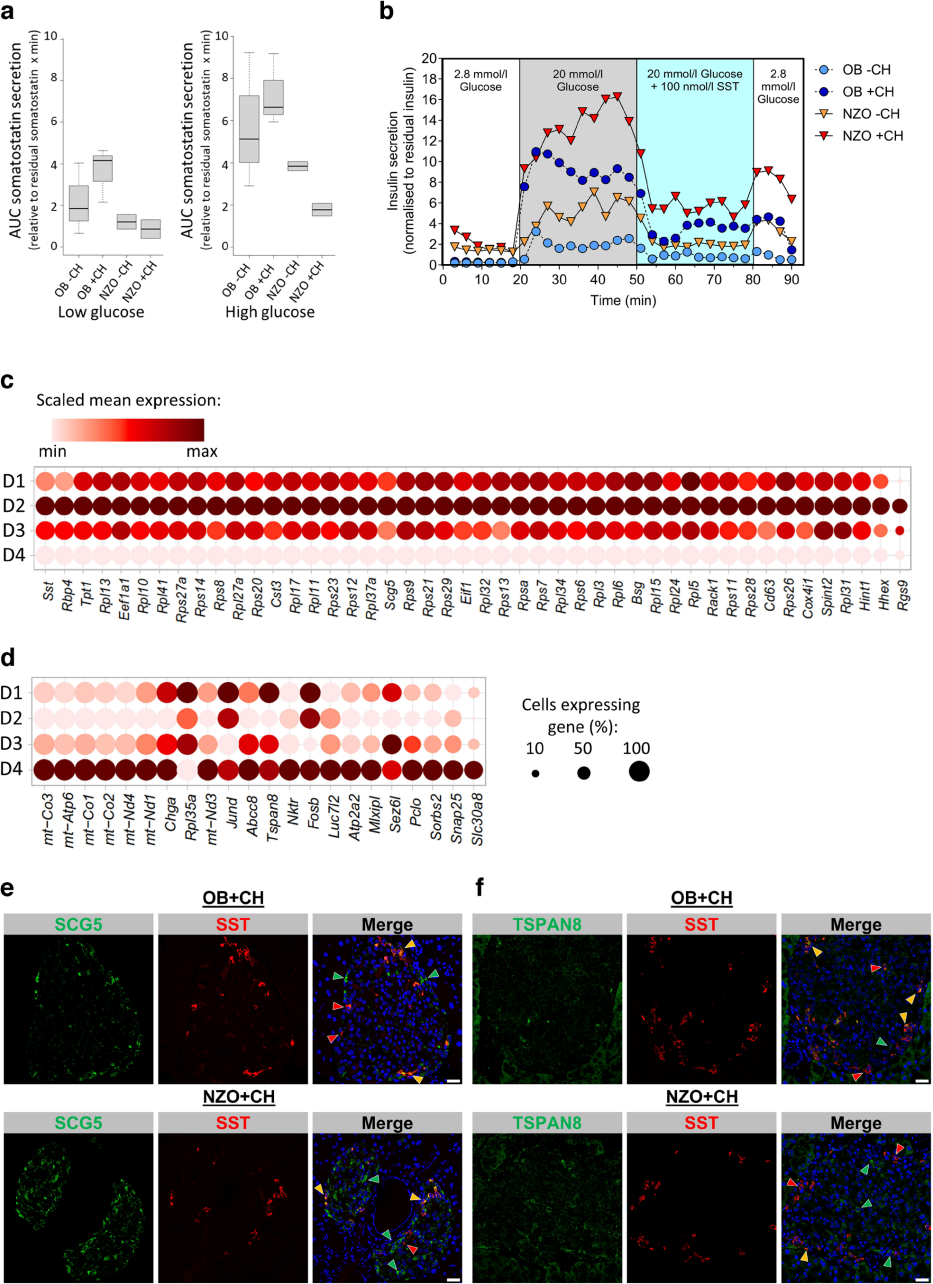
Lower somatostatin secretion from NZO mouse islets and DEGs of delta cell clusters D1/3/4 and D2. (**a**) Perifusion experiment of islets from OB and NZO mice fed with (+CH) and without (−CH) diabetogenic diet, showing AUC of somatostatin secretion at low (2.8 mmol/l) and high (20 mmol/l) glucose concentration (OB−/+CH: *n*=3, NZO−/+CH: *n*=2). (**b**) Perifusion experiment showing insulin secretion under low (2.8 mmol/l) and high (20 mmol/l) glucose, as well as the effects of inhibition by somatostatin (20 mmol/l glucose + 100 nmol/l somatostatin). Data are represented as mean ± SEM; OB−CH: *n*=3, OB+CH: *n*=5, NZO−/+CH: *n*=2. (**c**, **d**) Dot plot depicting expression of genes higher in either the D2 cluster (**c**; predominant in NZO mice) or the D1/3/4 clusters (**d**; predominant in OB mice). (**e**, **f**) Co-staining of pancreatic sections from OB +CH-fed mice and NZO +CH-fed mice for somatostatin and SCG5 (**e**) or somatostatin and TSPAN8 (**f**). Green arrowheads indicate SCG5/TSPAN8 single-positive cells; red arrowheads indicate somatostatin single-positive cells; yellow arrowheads indicate SCG5/TSPAN8 and somatostatin double-positive cells. Scale bar, 25 µm. SST, somatostatin

Delta cells play an important role as negative regulators of beta cell insulin and alpha cell glucagon release [42]. As we observed heterogeneity of delta cells in our mouse models, we measured somatostatin secretion via islet perifusion assay. Compared with OB mice, we observed reduced somatostatin secretion of islets from NZO −CH- and +CH-fed mice under both low and high glucose conditions. In contrast, islets from OB +CH-fed mice exhibited a further increase in somatostatin release compared with islets from OB −CH-fed mice and NZO mice, particularly under low glucose (Fig. 5a and ESM Fig. 5b). Despite the higher somatostatin secretion of islets from OB compared with NZO mice, there was no indication of insensitivity to somatostatin in either mouse strain, as the suppression of insulin secretion by somatostatin was comparable between groups (Fig. 5b). To link the transcriptome to the changes in somatostatin secretion, we compared the transcriptome of delta cell clusters D1, D3 and D4 (predominant in OB mice) with D2 (predominant in NZO mice). We identified 184 DEGs, of which 69 genes were also differentially expressed in human islets (ESM Table 2, ESM Fig. 3b); 47 displayed higher expression in NZO-enriched D2 cells (Fig. 5c) than in OB-enriched D1 cells. These included genes encoding for SCG5 and somatostatin and the transcriptional regulator gene *Hhex* [38], indicating a compensatory effect for the reduced somatostatin secretion of NZO mouse islets (Fig. 5a and ESM Fig. 5b). For the remaining 22 genes, expression was higher in OB-mouse-specific D1, D3 and D4 delta cell clusters (Fig. 5d) than in the D2 cluster. Among these were genes encoding for ATP binding cassette subfamily C member 8 (*Abcc8*), chromogranin A (*Chga*), piccolo presynaptic cytomatrix protein (*Pclo*), synaptosome associated protein 25 (*Snap25*) and tetraspanin 8 (*Tspan8*), which are involved in insulin secretion [43, 44] (Fig. 5d). Shared mechanisms between beta cell and delta cell exocytosis suggest that these genes might also affect somatostatin secretion. As a result, insulin hypersecretion of NZO mouse islets could be partially due to lower somatostatin levels, independent of the ability of somatostatin to suppress insulin secretion. Furthermore, we validated the changes in expression of *Scg5* and *Tspan8* at the protein level through histological analysis (Fig. 5e,f). As predicted, SCG5 was less abundant in delta cells of OB +CH-fed mice, as indicated by weaker co-staining with somatostatin compared with NZO mouse islets (Fig. 5e). Conversely, TSPAN8 was more abundant in delta cells of OB as opposed to NZO mice, demonstrated by a higher number of OB mouse cells co-stained with somatostatin (Fig. 5f). Thus, our data indicate that delta cell heterogeneity can be linked to somatostatin secretion in our mouse models.

### OB mouse islets display a higher abundance of macrophages than NZO mouse islets

**Fig. 6:**
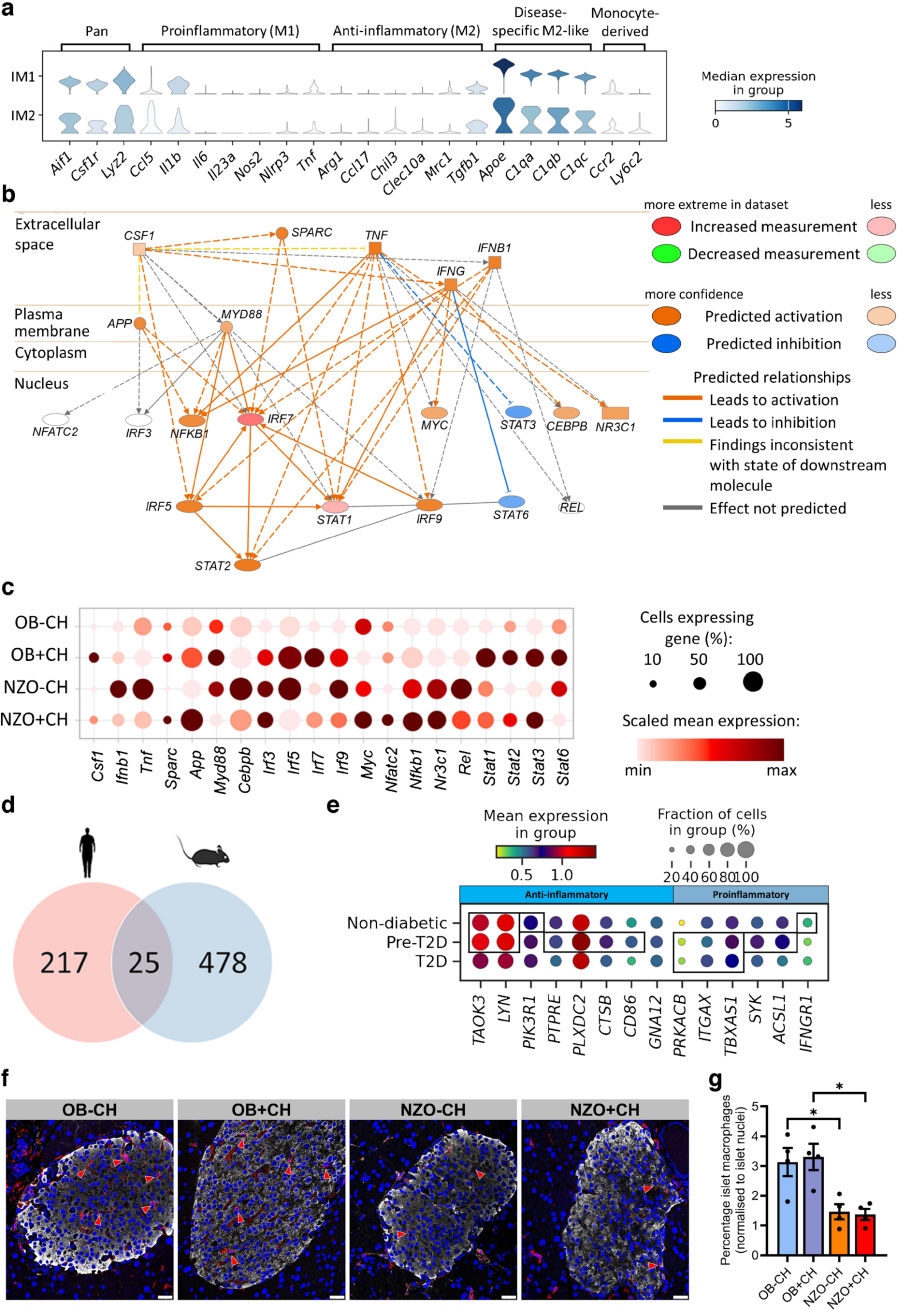
Functional annotation of unique gene expression patterns of anti- and proinflammatory macrophage clusters in mice and humans. (**a**) Stacked violin plot showing the expression of pan, pro- and anti-inflammatory, disease-specific and monocyte-derived macrophage marker genes in clusters IM1 and IM2. (**b**) Network constructed using ingenuity pathway analysis (IPA) upstream tools. Dashed lines indicate indirect relationships and solid lines indicate direct relationships. Genes highlighted in orange are putatively activated; genes highlighted in blue are putatively deactivated. The clear ovals indicate that genes could not be predicted by IPA. (**c**) Expression levels of genes that are regulated by IFN-γ in macrophages of OB and NZO mice fed with −CH or +CH. (**d**) Overlap of DEGs from human macrophage clusters (HM1 vs HM2, HM2 vs HM3 and HM1 vs HM3; false discovery rate [FDR] <0.01 and coefficient [coef] >0.05) with mouse macrophage clusters (IM1 vs IM2; FDR<0.01 and coef >0.2). (**e**) Expression level of anti- and proinflammatory genes in macrophages of human pancreatic islets from control individuals, people with prediabetes, and people with diabetes (*n*=11, 8 and 15, respectively). (**f**) Representative images of pancreatic sections from OB and NZO mice fed with −CH or +CH, stained for a macrophage marker (F4/80; red arrowheads) and insulin (INS). Scale bar, 25 µm. (**g**) F4/80-positive staining within islets (INS^+^ area) was counted and divided by the number of islet nuclei (DAPI). For each condition, several images (7–20) were counted (*n*=4). Data in (**g**) are presented as mean±SEM. *p<0.05 (one-way ANOVA with Šidák’s multiple comparisons test)

We next focused on islet macrophages. While cluster IM1 was similarly abundant in OB and NZO mouse islets, the number of IM2 cells increased only in islets from OB mice fed with +CH, and other immune cells were most abundant in OB islets under both −CH and +CH feeding (Fig. 1). Screening IM1 and IM2 for anti-inflammatory and proinflammatory markers revealed that these clusters displayed alterations in both, which precludes a definitive classification of polarisation state. Both clusters had a relative high expression of proinflammatory marker genes *Il1b*, *Ccl5* and *Tnf* (Fig. 6a). However, when compared with IM1, IM2 had at least a moderately higher number of cells expressing anti-inflammatory markers such as *Chil3*, *Ccl17* and *Clec10a* and fewer cells expressing M1 markers such as *Il1b*, *Il6* and *Tnf*, indicating less detrimental effects of IM2 on beta cells [45–47]. Since it is known that beta cells from OB mice are more protected from apoptosis than those from NZO mice [35], it can be speculated that a high abundance of islet macrophages and their expression and secretion of anti-apoptotic cytokines or other factors may contribute to the protection (Fig. 1).

To further characterise the differences between the three immune-cell clusters, the top ten marker genes were assessed, revealing a high expression of *Stat1* and *Sp100* in IM2 cells (ESM Fig. 6a), pointing to macrophage polarisation, differentiation [48] and changes in chromatin states (*Sp100*). In contrast to IM1 and IM2, immune-cell-enriched genes were more associated with endocrine pancreas (*Chga/b*, *Scg2/3* and *Resp18*) rather than macrophages. To understand which regulator might be responsible for sub-clustering of IM1 and IM2 cells, we performed Ingenuity upstream effector analysis of 503 DEGs. Among the top ten upstream regulators (ESM Table 3), the top five were IFN-γ, IL-4, colony stimulating factor 1 (CSF1), TNF and IL-10, which are some of the main signalling factors in macrophage polarisation [49]. This indicates that the two populations could differ in their effects on beta cells. In addition, the generated network (Fig. 6b) points to a shift towards activation of STAT and IFN regulatory factors, transcription factors known to be altered during macrophage polarisation and monocyte-to-macrophage differentiation [50].

In total, the network of IFN-γ consists of 21 nodes related to 20 genes, eight of which were expressed at higher levels in macrophages of OB mice vs 11 in NZO mice, indicating stronger proinflammatory effects in macrophages of NZO mice (Fig. 6c). The eight genes with greater expression in OB than in NZO macrophages included not only *Stat3* and *Stat6*, which are usually reduced by proinflammatory signalling via TNF and IFN-γ, but also *Stat1* and *Irf7*, which are rather induced by proinflammatory signalling and could indicate a higher sensitivity to IFN-γ (Fig. 6c). Overall, proinflammatory effects are linked to IM1 macrophages. While a classification of IM2 into M1- or M2-like macrophages is not possible due to indications for both pro- and anti-inflammatory signals, we speculate that those macrophages might be beta cell protective, due to expression of some anti-inflammatory markers as well as lower levels of proinflammatory markers when compared with IM1.

Furthermore, the number of macrophages in human islets was decreased [29] during type 2 diabetes progression (Fig. 3). Interestingly, the projection of macrophage clusters from humans to mice using scmap [51] revealed that most of the human macrophages were mapped to IM2 (ESM Fig. 6b). The comparison of DEGs in macrophages of both species identified 25 overlapping genes (Fig. 6d). Among those, eight were previously reported to have a rather anti-inflammatory effect (ESM Table 4) and were more highly expressed in macrophages of people without diabetes or with prediabetes (Fig. 6e). In contrast, six genes encoding for proinflammatory markers showed greater expression in macrophages of people with diabetes (Fig. 6e). However, the differences in expression in human islets were rather small. Therefore, the absolute decrease in macrophage number during type 2 diabetes development might be a more important factor in human type 2 diabetes pathogenesis.

Finally, we aimed to validate the observed differences in macrophage abundance histologically. Therefore, we performed immunostaining of F4/80 as a proxy for macrophages in a blinded manner, revealing that the proportion of staining in islets of diabetes-resistant OB mice was nearly double that in NZO mouse islets (Fig. 6f,g). These findings corroborate our results obtained from scRNA-seq data (Fig. 1).

### Cell–cell communication between macrophages and endocrine islet cells drives anti- and proinflammatory effects

**Fig. 7:**
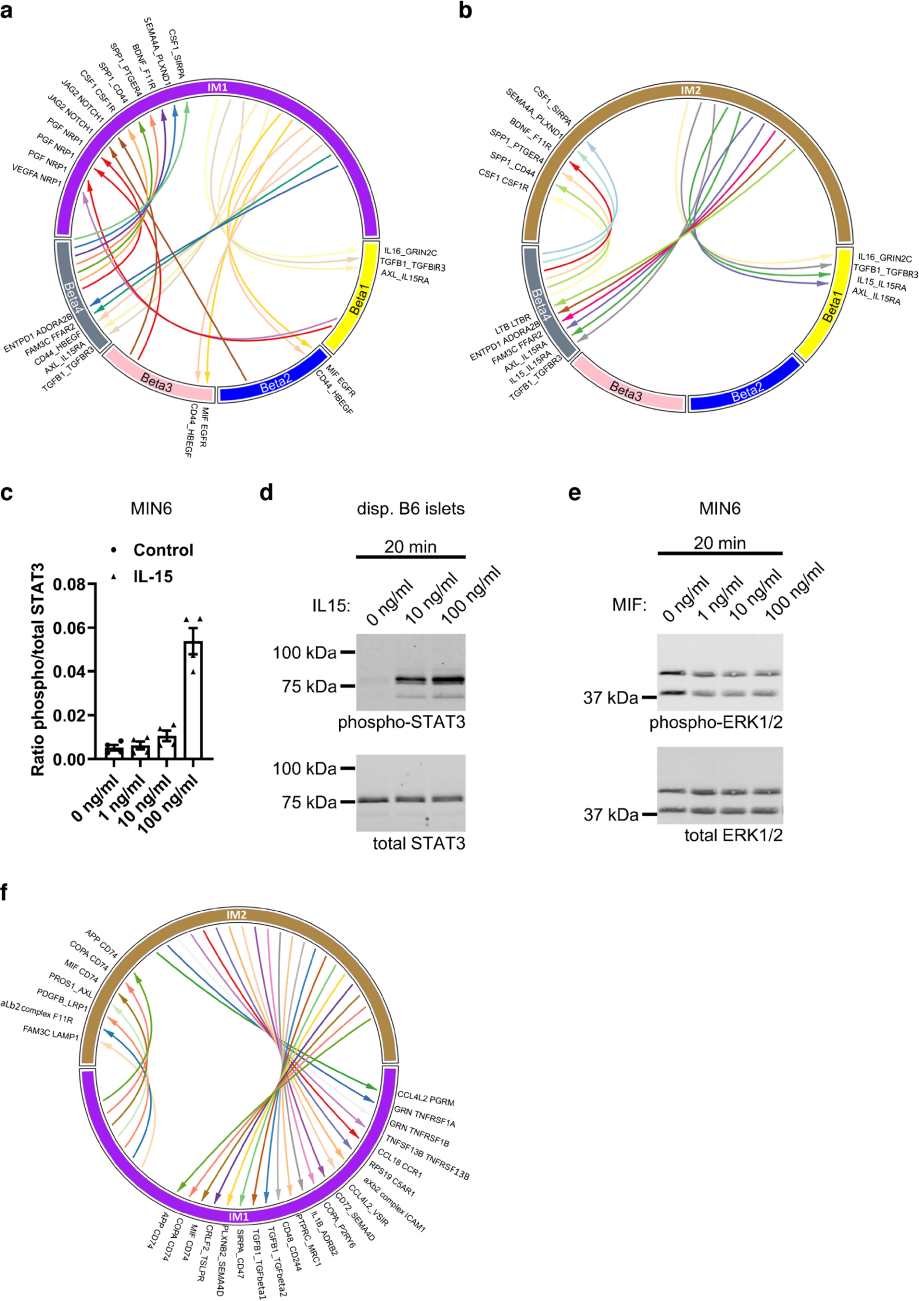
Effects of cell–cell communication between clusters of macrophages and beta cells. (**a**, **b**) Cell–cell communication of clusters IM1 (**a**) and IM2 (**b**) with different beta cell clusters. (**c**, **d**) Quantification of STAT3 phosphorylation in MIN6 cells (**c**, *n*=4) and western blot of dispersed islets (**d**, *n*=2) 20 min after treatment with IL-15. Data in (**c**) are presented as mean±SEM. (**e**) ERK1/2 phosphorylation in MIN6 cells treated with MIF for 20 min (*n*=2). (**f**) Cell–cell communication between the IM1 and IM2 clusters. ADORA2B, adenosine A2B receptor; ADRB2, adrenoceptor β2; aXb2, integrin α-X β2 complex; C5AR1, complement C5a receptor 1; CCL4L2, C-C motif chemokine ligand 4 like 2; CCL18, C-C motif chemokine ligand 18; CCR1, C-C motif chemokine receptor 1; CRLF2, cytokine receptor like factor 2; disp., dispersed; ENTPD1, ectonucleoside triphosphate diphosphohydrolase 1; FAM3C, metabolism-regulating signalling molecule C; FFAR2, free fatty acid receptor 2; GRIN2C, glutamate ionotropic receptor NMDA type subunit 2c; ICAM1, intercellular adhesion molecule 1; JAG2, jagged canonical notch ligand 2; LAMP1, lysosomal associated membrane protein 1; LTB, lymphotoxin β; LTBR, lymphotoxin β receptor; MRC1, mannose receptor C-type 1; NOTCH1, neurogenic locus notch homolog protein 1; NRP1, neuropilin-1; phospho, phosphorylated; PROS1, protein S; RPS19, ribosomal protein S19; PTPRC, protein tyrosine phosphatase receptor type C; TSLPR, cytokine receptor like factor 2; VEGFA, vascular endothelial growth factor A

Macrophages play a pivotal role in different tissues and diseases. In adipose tissue, for instance, infiltration of different macrophage types in the lean or obese state leads to anti- or proinflammatory effects [52], depending on the macrophage population (M1-/M2-like). Therefore, we were interested in the extent to which the identified macrophage clusters might participate in the adaptation or dysfunction of OB (Beta4) and NZO mouse beta cells (Beta2/3), respectively [24]. To gain insight into possible ligand–receptor interactions, we used CellPhoneDB [31] and identified an increase in cell–cell communication between both IM1 and IM2 in beta cells enriched in OB mouse islets (Beta4; Fig. 7a,b). Several predicted interactions of IM1 and IM2 ligands and receptors expressed in Beta1 (unstressed beta cells) and Beta4 cells were shared, including interactions that might be involved in the protection of beta cells from apoptosis (IL-15_IL15RA [IL-15 receptor subunit α], TGFB1_TGFBR3), and one receptor–receptor interaction (AXL [tyrosine-protein kinase receptor UFO]_IL15RA; Fig. 7a,b). Interestingly, NicheNet [32] also identified STAT3 as a downstream target of IL-15 or IL15RA (ESM Fig. 7a), and expression profiling in islet macrophage and beta cell clusters revealed that this might be one of the most promising ligand–receptor pairs to test (ESM Fig. 7b, c).

To test the impact of potential macrophage ligands on beta cell function, MIN6 cells and primary islets were treated with IL-15 and STAT3 activation was measured. We detected a dose-dependent increase in STAT3 phosphorylation (Fig. 7c,d), which might antagonise mothers against decapentaplegic homolog 3 (SMAD3) and TGF-β signalling in OB-enriched Beta4 cells [53].

IM1 cells also displayed elevated expression levels of MIF and CD44, which were predicted to interact with EGF receptor (EGFR) and proheparin-binding EGF-like growth factor (HBEGF) receptors of cells assigned to the Beta2/3 cluster (beta cells enriched in NZO +CH-fed mice) (Fig. 7a). This is contradictory to what we would expect, as EGFR and HBEGF are described as being positive regulators of cell cycle and proliferation [54], which is not induced in NZO mouse islets under +CH feeding. However, MIF is also known to mediate beta cell dysfunction [55] and therefore might be a driver of this in NZO mice. Treatment of MIN6 cells with MIF decreased phosphorylation of ERK1/2, confirming our hypothesis that it might be linked to detrimental effects in beta cells (Fig. 7e).

CellPhoneDB also predicted extended cell–cell communication between OB mouse beta cells (Beta4) and IM1 and IM2 cells via various ligand–receptor pairs (CSF1_CSF1R, secreted phosphoprotein 1 [SPP1]_PTGER4 [prostaglandin E2 receptor EP4 subtype], brain-derived neurotrophic factor [BDNF]_F11R [junctional adhesion molecule A], SPP1_CD44 [CD44 antigen] and CSF1_SIRPA [signal regulatory protein α]; Fig. 7a,b). Of note, secretion of CSF1 contributes to a microenvironment that is linked to organ repair in a range of disease models [56], is related to macrophage differentiation and survival [11], and was also one of the upstream regulators identified via the Ingenuity tools (Fig. 6b). These observations indicate that OB mouse beta cells are not only protected via a high abundance of macrophages but also participate in the induction of protective effects via crosstalk with IM1 and IM2 cells. Similarly, we investigated the interactions of the macrophage clusters (IM1 and IM2) with alpha cells (A1–A4). Our findings indicated that the A3 cluster (more prominent in OB mouse islets) communicates with both islet macrophage clusters (e.g. via progranulin GRN_TNFRSF1A [TNF receptor superfamily member 1A] or plexin B2 PLXNB2_SEMA4C [semaphorin-4C]), whereas no interaction was detected for the A2 cluster highly abundant in NZO mouse islets (ESM Fig. 8a, b). GRN is the granulin precursor, a secreted growth factor that binds to TNF receptors, which for instance inhibits inflammatory arthritis [57]. PLXNB2 is a transmembrane receptor for SEMA4C and participates in cell migration via activation of RhoA [58]. Similar to beta cells, a putative interaction of IM2 via IL15_IL15RA was only found in OB mouse alpha cells (A3).

In addition, we detected a large number of putative communications between macrophages and delta cells. It appears that IM1 mainly interacts with the D1 and D4 clusters, which are both more abundant in OB mouse islets. In the opposite direction, D1–D4 ligands may also bind to receptors expressed in IM1 and IM2 cells (ESM Fig. 8c, d).

Finally, cell–cell communication between the IM1 and IM2 clusters was evaluated in order to indicate how the different macrophages could influence each other. CellPhoneDB predicted a higher number of ligand–receptor interactions of IM2 with IM1 cells than vice versa (Fig. 7f). Nevertheless, both macrophage clusters were predicted to activate the major histocompatibility complex, class II invariant chain (CD74) receptor via amyloid β precursor protein (APP), coatomer protein complex subunit α (COPA) and MIF (Fig. 7f). Activation of CD74 via MIF is related to tumour growth [59] and might mediate effects on proliferation in islet macrophages. Overall, our analysis indicates intensive cell–cell communication both within macrophage clusters and between islet macrophages and beta cells.

### Similarity of macrophages in zebrafish, mice and humans

To underline our assumption that islet macrophages are related to beta cell health, we tested changes in macrophage populations in a model of beta cell regeneration, which was recently described in zebrafish [30]. Beta cell regeneration was induced via metronidazole treatment, resulting in rapid and complete beta cell apoptosis. At 14 dpa, zebrafish had fully restored glucose homeostasis with insulin-expressing beta/delta1 hybrid cells (Delta1). As our mouse data reflect early transcriptome alterations during the onset of hyperglycaemia, we investigated zebrafish islets at 0 and 2 dpa. Our data analysis revealed clusters similar to those reported in the original study by Singh et al [30] (ESM Fig. 9a). Re-clustering of zebrafish immune cells (ESM Fig. 9b) resulted in three macrophage clusters (Immune_1, 2 and 3). The full characterisation of zebrafish macrophages can be found in ESM Results. To compare the different macrophage populations found in zebrafish, mouse and human islets, we first determined the top 250 marker genes for each cluster in each species and then compared the significantly enriched pathways. Hierarchical clustering of these pathways grouped the macrophage clusters such that IM1 and IM2 (mouse) were most similar to HM1 (human) and Immune_1 (zebrafish). Interestingly, Immune_1 cells doubled during beta cell regeneration in zebrafish and HM1 cells were diminished during type 2 diabetes development in human islets (ESM Fig. 10), suggesting that these cells have a net positive effect and their loss is detrimental to islet health. Overall, our analysis suggests a similar anti-inflammatory effect of macrophages in islets of both vertebrate model systems and humans, which may contribute to beta cell regeneration or health.

## Discussion

Our data indicate that cell heterogeneity is an important aspect in the pathogenesis of type 2 diabetes, not only for beta cells but also for alpha cells, delta cells and macrophages in islets of humans and mice. Interestingly, macrophages appear to play a major role via cell–cell communication with all endocrine cells, while different delta cell clusters and their transcriptional patterns affect somatostatin secretion.

To date, most scRNA-seq studies have focused on the role of beta cells in type 2 diabetes [5, 24, 25]. Two studies already established a link between beta cell and alpha cell heterogeneity and type 2 diabetes pathogenesis [26, 60]. Here, we used the NZO mouse as a well-controlled and balanced model of hyperglycaemia to explore to what extent transcriptional changes in other islet cell types can be associated with type 2 diabetes pathophysiology. Previously, we confirmed the impact of beta cell heterogeneity on type 2 diabetes using this model [24]. In the present study, we found indications that altered mitochondrial function is linked to alpha cell heterogeneity in type 2 diabetes (Fig. 4) and that transcriptional delta cell heterogeneity associates with changes in somatostatin secretion (Fig. 5). It would be informative to know if specific alpha cell populations group together or if they are distributed randomly in the islets. However, since none of the transcripts exhibit an exclusive expression in any of the alpha cell clusters, we were not able to answer this question by staining of the corresponding proteins. Furthermore, several cytokines (IL-15, TGFB1, and MIF) released by islet macrophages might influence beta cell health. This was indicated by expression patterns that point towards specific cell–cell communications in islets of diabetes-resistant OB mice (Fig. 7a,b), which may affect downstream targets like *Id4*, *Ltbp4* and *Stat3*. TGFβ1 secretion by M2-like macrophages has positive effects on beta cells via Smad signalling [56]. We hypothesise that putative effects of TGFβ-signalling might be mediated via phosphorylation of STAT3 [53], which in turn might prevent beta cell decline [61]. As OB mice are leptin-deficient, we propose a leptin-independent mechanism of STAT3 activation, which is mediated via macrophages in this diabetes-resistant mouse strain. In fact, an activation of STAT3 was induced by IL-15 treatment of MIN6 cells and dispersed islets. Additionally, there was evidence that beta cells of OB mice might support anti-inflammatory functions of macrophages via secretion of CSF1, which binds to CSF1R in macrophages [56]. Notably, we identified several other putative interactions: SPP1_PTGERA, SPP1_CD44, BDNF_F11R, and CSF1_SIRPA that appeared only between beta cells of OB mice (Beta4) and both IM1 and IM2 macrophages. Several roles related to macrophages have been described for these two ligands. *Spp1*-knockout mice exhibited a lower number of macrophages in gonadal adipose tissue [62], possibly explaining why NZO mouse islets exhibit fewer macrophages than OB mouse islets. BDNF suppresses proinflammatory cytokine secretion in macrophages [63] and might be another driver of macrophage polarisation to a more anti-inflammatory phenotype. Interestingly, beta cell regeneration data from zebrafish also indicate that macrophages undergo transcriptional changes towards an anti-inflammatory type. This suggests on the one hand that those macrophages trigger pathways that support beta cell survival when blood glucose concentration increases and on the other hand indicates that macrophages might be able to activate a transcriptional program that induces proliferation or trans-differentiation, both aspects that can be impaired in human type 2 diabetes. In fact, applying clustering of non-beta cells from scRNA-seq data from human islets identified three clusters of islet macrophages with a decreased abundance during the progression of prediabetes and type 2 diabetes. Additionally, anti-inflammatory macrophage genes were slightly higher expressed in islets of non-diabetic individuals than in donors with diabetes.

However, our study has several limitations, primarily due to its reliance on transcriptomics, with validation at the protein level only through selected staining and not through untargeted approaches. With advances in technology, future work will include such proteomics analyses. By extension, cell-type composition inferred from transcriptomics may also not accurately reflect true cell composition, as demonstrated in our previous study [10]. Here, we specifically validated our transcriptomics finding of higher macrophage abundance in islets of OB compared with NZO mice via immunostaining. In contrast, several human studies revealed an increase of macrophages in type 2 diabetes pathogenesis based on immune straining [52, 53]. Furthermore, those studies revealed a relative loss of beta cells, an increase of alpha cells, and no changes in delta cells in islets of people with type 2 diabetes compared with control individuals. Those findings are in line with our single-cell transcriptomics analyses of human islets. However, our mouse single-cell transcriptomics reveal a loss of alpha cells and an increase of beta cells with diabetogenic diet independent of mouse strain. This may be explained by the very early disease state triggered by 2 days of diet-induced glucolipotoxic stress. At this stage, beta cells of OB and NZO mice undergo the initial changes that lay the foundation for subsequent compensation or decompensation, respectively, including an increase in beta cell mass in OB mouse beta cells and gradual apoptosis in NZO mouse beta cells. Of note, the compensatory response of OB mouse beta cells may indicate that IM2 cells, predominantly abundant in the islets of this diabetes-resistant strain, contribute to a transcriptional program that supports beta cell homeostasis, proliferation and, thus, eventual escape from the glucolipotoxic stresses on the islet. To further elucidate the impact of islet cell heterogeneity of different cell types on type 2 diabetes, we mapped DEGs of mouse islet cells to data from human type 2 diabetes GWAS. Interestingly, the human type 2 diabetes risk gene *SUMO2*, which is specifically associated with alpha cell heterogeneity, was earlier linked to mitochondrial dysfunction in beta cells. Deletion of its conjugating enzyme sentrin-specific protease 2 (SENP2) in mice increased the small ubiquitin-like modifier 2/3 conjugation of mitochondrial fission protein dynamin-related protein 1 (DRP1) resulting in impaired beta cell secretion [64]. Therefore, alterations of *Sumo2* in alpha cells could affect alpha cell glucagon secretion via similar mechanisms and promote type 2 diabetes pathogenesis [65].

Mapping of type 2 diabetes GWAS genes to specific cells also revealed that macrophages and delta cells had a higher number of differentially expressed type 2 diabetes risk genes than pancreatic polypeptide and alpha cells. The identification of *Hhex* in delta cells, one of the rare cases of type 2 diabetes risk genes in non-beta cells, highlights the value of our translational approach. Another interesting gene that might link delta cell heterogeneity to the observed differences in delta cell abundance might be *Ache* (encoding acetylcholinesterase), a gene linked to beta cell apoptosis [66] and shown here to be expressed at higher levels in NZO compared with OB mouse delta cells. This potentially suggests higher delta cell apoptosis in NZO mice than in OB mice, consistent with a significantly lower number of delta cells in these mice. Additionally, we associated transcriptional heterogeneity with alterations in somatostatin secretion. For example, we identified lower expression of *Chga* and *Tspan8* and lower abundance of TSPAN8 protein in delta cells of NZO mice. *Tspan8* and *Chga* are both associated with insulin secretion [43, 44], with the latter also related to vesicle storage [43]. Therefore, both genes might also be important for somatostatin secretion and could partially explain higher somatostatin secretion in OB mice under +CH feeding. Despite a lower somatostatin secretion, delta cells (D2) of NZO mice showed higher expression levels of somatostatin and *Hhex*, potentially as a compensatory effect. However, we believe that a set of DEGs between D1/D3/D4 and D2 is responsible for the observed higher somatostatin secretion in OB mice.

Overall, our explorative bioinformatic approach using mouse and human islets complemented with experiments makes a strong case for a more in-depth analysis of non-beta cell heterogeneity, particularly of delta cells and macrophages, to refine molecular mechanisms involved in type 2 diabetes pathogenesis.

## Supplementary Information

Below is the link to the electronic supplementary material.

ESM (PDF 2303 KB)

## Data Availability

scRNA-seq data from the previous study are available in gene expression omnibus under gene accession number GSE159211 (https://www.ncbi.nlm.nih.gov/geo/query/acc.cgi?acc=GSE159211).

## Abbreviations

−CH: Carbohydrate-free high-fat diet
+CH: Carbohydrate-containing diabetogenic diet
BDNF: Brain-derived neurotrophic factor
CD44: CD44 antigen
CD74: Major histocompatibility complex, class II invariant chain
CSF1: Colony stimulating factor 1
DEG: Differentially expressed gene
dpa: Days post ablation
EGFR: EGF receptor
F11R: Junctional adhesion molecule A
GEO: Gene Expression Omnibus
GRN: Progranulin
GWAS: Genome-wide association study
HBEGF: Proheparin-binding EGF-like growth factor
IL15RA: IL-15 receptor subunit α
IM1: Islet macrophage cluster 1
IM2: Islet macrophage cluster 2
MIF: Macrophage migration inhibitory factor
NZO: Diabetes-susceptible New Zealand Obese (mice)
OB: Diabetes-resistant B6.V.*Lep*^ob/^^ob^ (mice)
PLXNB2: Plexin B2
SCG5: Secretogranin V
scRNA-seq: Single-cell RNA sequencing
SEMA4C: Semaphorin-4C
SIRPA: Signal regulatory protein α
SPP1: Secreted phosphoprotein 1
STAT3: Signal transducer and activator of transcription 3
TSPAN8: Tetraspanin 8
