# Supplementary material for: Transcriptomic heterogeneity of non-beta islet cells is associated with type 2 diabetes development in mouse models
Source: Diabetologia. 2024 Nov 7;68(1):166–85. doi: 10.1007/s00125-024-06301-6 (PMC11663180; doi:10.1007/s00125-024-06301-6)
Supplement: Supplementary file 1 — ESM (PDF 2303 KB) [file 125_2024_6301_MOESM1_ESM.pdf]

ESM Results:

Role of macrophages in zebrafish beta-cell regeneration

Interestingly, the abundance of immune cell cluster 1 doubled (ESM Fig. 9b) two days after beta-cell ablation and exhibited increased expression levels of anti-inflammatory marker genes (ESM Fig. 9c; *apoeb*, *c1qa/b* and *ctsc*), and of markers known to indicate MΦ2-like polarisation (*lgmn*, *cd74a* and *havr1*) [1–3] (ESM Fig. 9d). This was comparable to the observation of IM2 cells in OB mice 2 days after carbohydrate feeding. In contrast, the marker genes of zebrafish Immune_3 were more related to a pro-inflammatory phenotype, e.g. via expression of *epas1b* and *sox7*. Cells belonging to the Immune_2 cluster did not exhibit particular signature genes, but displayed an expression pattern closer to Immune_1 than Immune_3. To further compare the mouse and zebrafish datasets, we screened zebrafish immune cell clusters for receptors that mediate MΦ1 to MΦ2 polarisation, as well as ligands facilitating protective effects on beta cells in mice. Based on homology and detection limits, we were only able to translate 8 out of 13 mouse candidates to the zebrafish dataset. However, expression values of ligands in Immune_1 and Immune_3 best reflected expression values of those ligands in anti-inflammatory islet macrophages of mice (ESM Fig. 9e). In addition, receptor expression levels of Immune_2 best reflected a set of receptors that promote a switch from inflammatory to anti-inflammatory macrophages (ESM Fig. 9e). Those similar expression patterns of ligands (*il15*, *mif*) and receptors (*csfr1a/b*) provide another line of evidence for shared molecular mechanisms.

References

1. Sun SG, Guo JJ, Qu XY, et al (2022) The extracellular vesicular pseudogene LGMNP1 induces M2-like macrophage polarization by upregulating LGMN and serves as a novel promising

predictive biomarker for ovarian endometriosis recurrence. Hum Reprod 37(3):447–465. https://doi.org/10.1093/HUMREP/DEAB266

2. Orecchioni M, Ghosheh Y, Pramod AB, Ley K (2019) Macrophage polarization: Different gene signatures in M1(Lps+) vs. Classically and M2(LPS-) vs. Alternatively activated macrophages. Front Immunol 10(MAY):1084. https://doi.org/10.3389/FIMMU.2019.01084/BIBTEX

3. Gundra UM, Girgis NM, Ruckerl D, et al (2014) Alternatively activated macrophages derived from monocytes and tissue macrophages are phenotypically and functionally distinct. Blood 123(20):e110–e122. https://doi.org/10.1182/BLOOD-2013-08-520619

ESM Table 1. Cellular localisation and function of genes unique for different alpha-cell clusters.

ID	Location	Type	Unique expression in
Actg1	Cytoplasm	Others	A2
Eif1	Other	Others	A2
Ociad2	Cytoplasm	Others	A2
Pcsk1n	Extracellular Space	Others	A2
Tm4sf4	Plasma Membrane	Others	A2
Upk3a	Cytoplasm	Others	A2
Cst3	Extracellular Space	Cell death	A2
Ddx5	Nucleus	Enzyme	A2
Gnas	Plasma Membrane	Enzyme	A2
Top1	Nucleus	Enzyme	A2
Fkbp5	Nucleus	Protein folding	A2
Hspa1b	Cytoplasm	Protein folding	A2
Hspa8	Cytoplasm	Protein folding	A2
Fosb	Nucleus	Transcriptional regulator	A2
Rbm39	Nucleus	Transcriptional regulator	A2
Sox4	Nucleus	Transcriptional regulator	A2
Zbtb20	Nucleus	Transcriptional regulator	A2
Rpl12	Nucleus	Ribosomal	A2
Rpl13	Nucleus	Ribosomal	A2
Rpl14	Cytoplasm	Ribosomal	A2
Rpl15	Cytoplasm	Ribosomal	A2
Rpl22l1	Cytoplasm	Ribosomal	A2
Rpl34	Cytoplasm	Ribosomal	A2
Rpl38	Cytoplasm	Ribosomal	A2
Rps12	Cytoplasm	Ribosomal	A2
Rps16	Cytoplasm	Ribosomal	A2
Rps21	Cytoplasm	Ribosomal	A2
Rps27	Cytoplasm	Ribosomal	A2
Rps3	Cytoplasm	Ribosomal	A2
Rps4x	Cytoplasm	Ribosomal	A2
Rps5	Cytoplasm	Ribosomal	A2
Rps7	Cytoplasm	Ribosomal	A2
Scg2	Extracellular Space	Cytokine	A2
Wnt4	Extracellular Space	Cytokine	A2
Srsf3	Nucleus	Splicing	A2
Dctn3	Nucleus	Others	A3
Spint2	Extracellular Space	Others	A3
mt-Atp6	Cytoplasm	Mitochondrial	A3
mt-Co1	Cytoplasm	Mitochondrial	A3

mt-Co2	Cytoplasm	Mitochondrial	A3
mt-Co3	Cytoplasm	Mitochondrial	A3
mt-Nd1	Cytoplasm	Mitochondrial	A3
mt-Nd3	Cytoplasm	Mitochondrial	A3
mt-Nd5	Cytoplasm	Mitochondrial	A3
Rpl29	Cytoplasm	Ribosomal	A3
Bambi	Plasma Membrane	Others	A4
Higd1a	Cytoplasm	Others	A4
Mt1	Cytoplasm	Others	A4
Spcs2	Cytoplasm	Others	A4
Tmsb4x	Cytoplasm	Others	A4
Tspan8	Plasma Membrane	Others	A4
Castor2	Cytoplasm	Protein-binding	A4
Gcg	Cytoplasm	Protein-binding	A4
Selenok	Cytoplasm	Protein-binding	A4
Ostc	Cytoplasm	Enzyme	A4
Pappa2	Extracellular Space	Peptidase	A4
Pcsk2	Extracellular Space	Peptidase	A4
Rpl17	Cytoplasm	Ribosomal	A4
Rpl18a	Cytoplasm	Ribosomal	A4
Rpl21	Cytoplasm	Ribosomal	A4
Rpl37a	Cytoplasm	Ribosomal	A4
Rpl41	Cytoplasm	Ribosomal	A4
Rpl9	Nucleus	Ribosomal	A4
Rplp1	Nucleus	Ribosomal	A4
Rps20	Cytoplasm	Ribosomal	A4
Rps24	Cytoplasm	Ribosomal	A4
Rps6	Cytoplasm	Ribosomal	A4
Rps8	Cytoplasm	Ribosomal	A4
Slc7a2	Plasma Membrane	Transporter	A4
Wnk3	Plasma Membrane	Kinase	A4

ESM Table 2. Genes differentially expressed in delta-cell clusters of human and mouse islets.

Primerid	Coef	FDR
ABCC8	0.48	5.13E-03
ATP2A2	0.38	3.00E-03
BSG	-0.27	8.78E-04
CD63	-0.43	2.67E-05
CHGA	-1.30	9.38E-16
COX4I1	-0.30	2.67E-05
CST3	-0.36	7.29E-08
EEF1A1	-0.28	1.07E-04
EGR1	-1.51	9.76E-32
EIF1	-0.35	3.25E-06
FOSB	-0.64	4.52E-20
HINT1	-0.23	9.62E-03
JUND	-1.00	6.65E-13
LUC7L2	0.74	3.98E-04
MLXIPL	0.92	2.41E-09
MT-ATP6	1.00	1.30E-68
MT-CO1	1.49	4.69E-65
MT-CO2	0.74	1.71E-67
MT-CO3	1.56	1.06E-62
MT-ND1	0.92	1.37E-29
MT-ND3	1.73	1.87E-40
MT-ND4	1.19	2.44E-62
NKTR	1.24	7.99E-06
PCLO	0.83	1.66E-09
RACK1	-0.82	6.97E-08
RBP4	-0.62	1.35E-05
RGS9	0.42	1.75E-03
RPL10	0.36	4.78E-03
RPL11	-0.37	1.73E-09
RPL13	-0.21	3.00E-03
RPL13	-0.30	2.11E-07
RPL15	-0.31	1.34E-04
RPL17	-0.35	7.71E-08
RPL24	-0.27	1.23E-04
RPL27A	-0.28	2.36E-06
RPL3	-0.36	2.11E-07
RPL31	-0.58	4.84E-03
RPL32	-0.25	1.02E-03
RPL34	0.33	9.29E-03
RPL35A	-0.25	9.23E-04
RPL37A	-0.31	2.01E-06
RPL41	-0.34	6.27E-11
RPL5	-0.34	8.32E-06
RPL6	-0.24	1.52E-05
RPS11	-0.24	2.69E-03
RPS12	-0.35	5.47E-10
RPS13	-0.21	1.11E-04
RPS14	-0.32	1.85E-09
RPS20	-0.41	2.41E-09
RPS21	-0.29	8.86E-05
RPS23	-0.23	1.14E-05
RPS26	-1.00	9.04E-03
RPS27A	-0.39	6.26E-10
RPS28	-0.59	1.16E-03
RPS29	-0.38	3.70E-08
RPS6	-0.42	2.16E-08
RPS7	-0.30	3.82E-04
RPS8	-1.17	2.04E-03
RPS9	-0.33	9.21E-06
RPSA	-1.20	5.47E-05
SCG5	0.91	2.01E-11
SEZ6L	0.23	6.36E-03
SLC30A8	0.86	6.91E-17
SNAP25	0.40	1.42E-03
SORBS2	0.49	2.67E-05
SPINT2	0.22	4.52E-03
SSR4	0.90	7.74E-06
SST	-0.88	6.37E-16
TPT1	-0.39	1.64E-12

ESM Table 3. Top 20 upstream regulator identified via IPA-tools.

Upstream regulator	Molecule type	Activation z-score	B-H corrected p-value	Target molecules
IFNG	cytokine	3.33	8.62E-66	112
IL4	cytokine	-1.87	8.35E-58	113
CSF1	cytokine	1.43	4.72E-42	56
TNF	cytokine	1.40	2.12E-36	65
IL10	cytokine	0.25	1.52E-33	53
IRF7	transcription regulator	5.48	2.33E-33	38
CITED2	transcription regulator	-3.92	2.33E-33	44
IRF3	transcription regulator	5.43	1.48E-32	34
TREX1	enzyme	-2.62	7.49E-31	30
PLCG2	enzyme	-2.40	4.03E-30	28
APP	other	2.11	2.86E-28	40
IL27	cytokine	3.91	7.03E-26	33
GRN	growth factor	2.56	9.52E-26	28
STAT3	transcription regulator	-2.24	6.63E-25	36
IL2	cytokine	0.70	6.63E-25	56
NR1H3	ligand-dependent nuclear receptor	-0.95	5.35E-24	32
STAT6	transcription regulator	-2.90	1.56E-23	42
IL21	cytokine	2.83	1.35E-22	30
CD40LG	cytokine	1.29	1.82E-22	41
BHLHE40	transcription regulator	1.59	1.07E-20	39

ESM Table 4. Literature indicating relevance for anti- or proinflammatory influences on macrophages.

Gene	Full name	Effect	PubmedID	Author	Year
ACSL1	acyl-CoA synthetase long chain family member 1	Pro-inflammatory	37416456	Al-Rashed	2020
CD86	CD86 molecule	Anti-inflammatory	30576000	Wang	2019
CTSB	cathepsin B	Anti-inflammatory	32927704	Oelschlaegel	2020
GNA12	G protein subunig alpha 12	Anti-inflammatory	37426201	Yu	2023
IFNGR1	interferon gamma receptor 1	Pro-inflammatory	37271317	Siebler	2023
ITGAX/CD11c	integrin subunit alpha X	Pro-inflammatory	34910922	Zhenghai	2021
LYN	LYN proto-oncogene Src family tyrosine kinase	Anti-inflammatory	29263906	Li	2016
PIK3R1	phosphoinositide-3-kinase regulatory subunit 1	Anti-inflammatory	22698915	McCurdy	2012
PLXDC2	plexin domain containing 2	Anti-inflammatory	32661418	Tubau-Juni	2020
PRKACB	protein kinase cAMP-activated catalytic subunit beta	Pro-inflammatory	32402274	YiRang	2020
PTPRE	protein tyrosine phsophatase receptor type E	Anti-inflammatory	31844669	Xiao	2023
SYK	spleen associated tyrosine kinase	Pro-inflammatory	25045209	Yi	2014
TAOK3	TAO kinase 3	Anti-inflammatory	37400834	Poirier	2023
TBXAS1	thromboxane A synthase 1	Pro-inflammatory	36849064	Ruijie	2023

ESM Figures

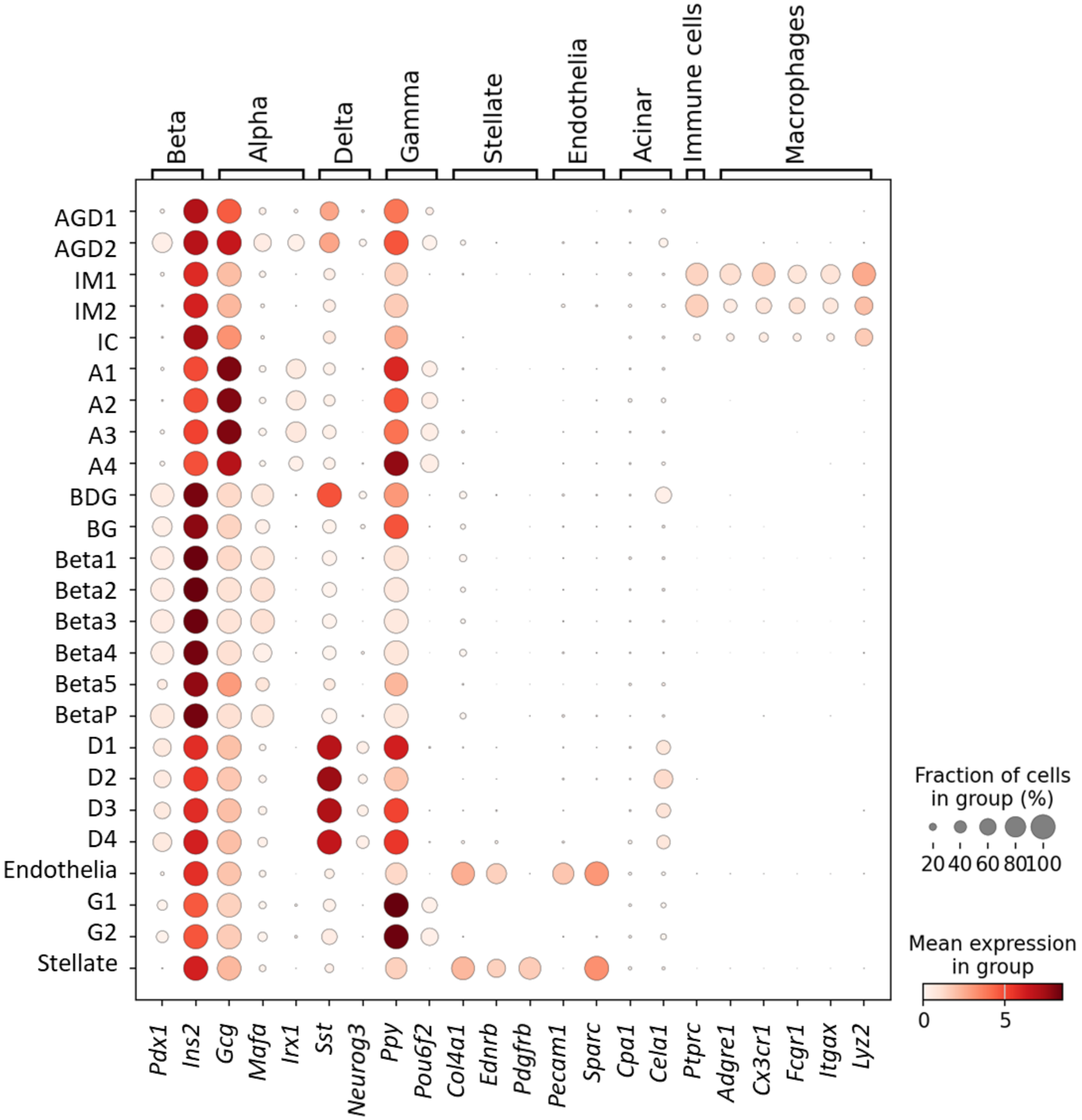

ESM Fig. 1. Dot plot demonstrating expression levels in identified clusters of marker genes for specific endocrine and exocrine pancreatic islet cell types as described in the literature.

a

b

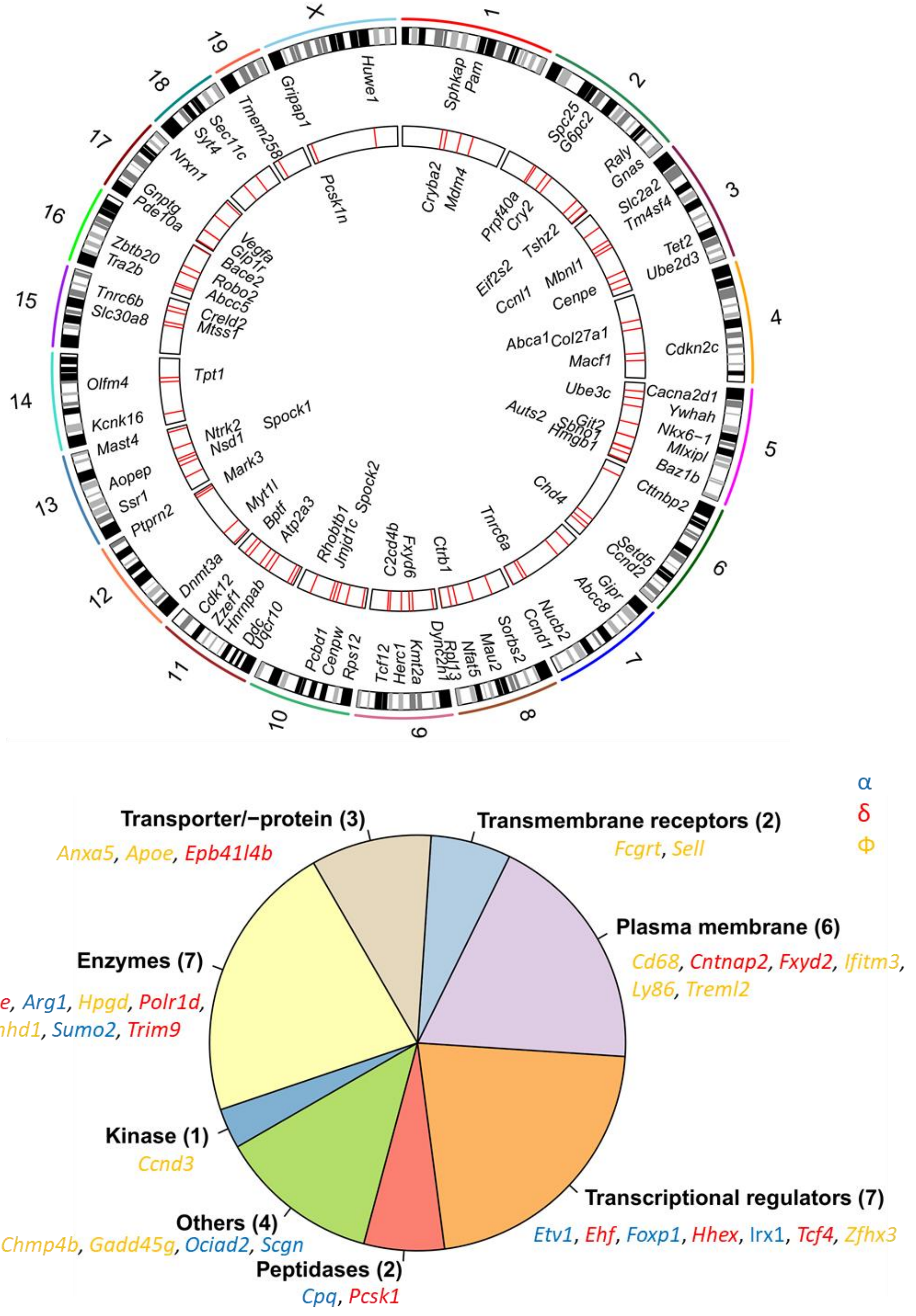

ESM Fig. 2. Classification of human T2D risk genes.

(**a**) Overlap of human T2D risk genes with all cell types. (**b**) Functional classification of T2D risk genes unique for one specific non-beta islet cell type. Genes highlighted in blue are unique for alpha cells, in red for delta cells, and yellow for macrophages.

a

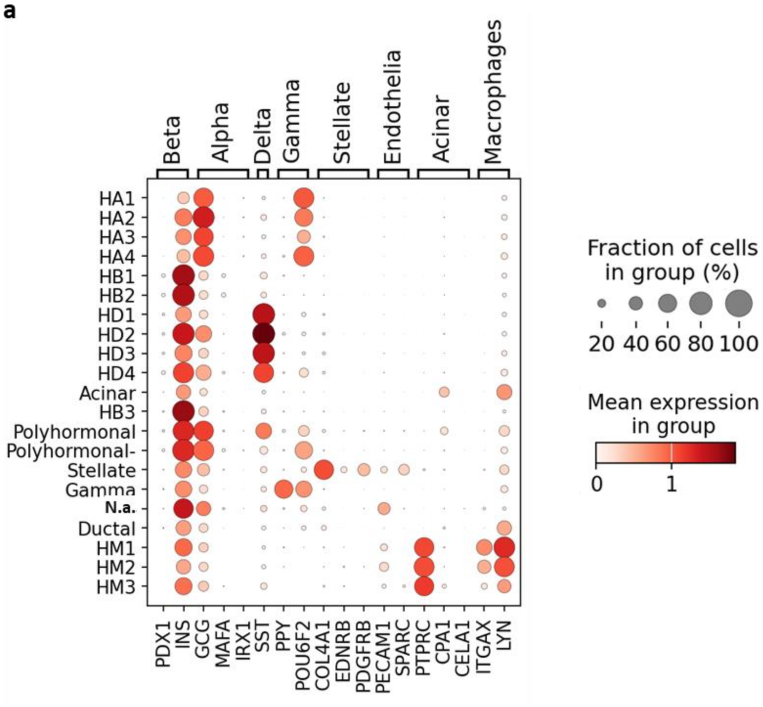

b

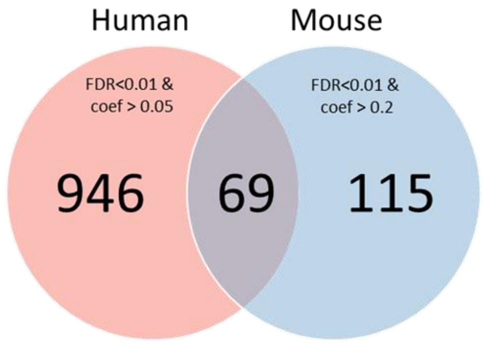

ESM Fig. 3. Marker gene expression in human islets and comparison of human and mouse delta cells.

(**a**) Dot plot showing expression levels in identified clusters of marker genes for specific endocrine and exocrine pancreatic islet cell types of human islets. (**b**) Venn diagram displaying the overlap of differentially expressed genes in delta cells of human and mouse islet. FDR: false discovery rate, coef: coefficient

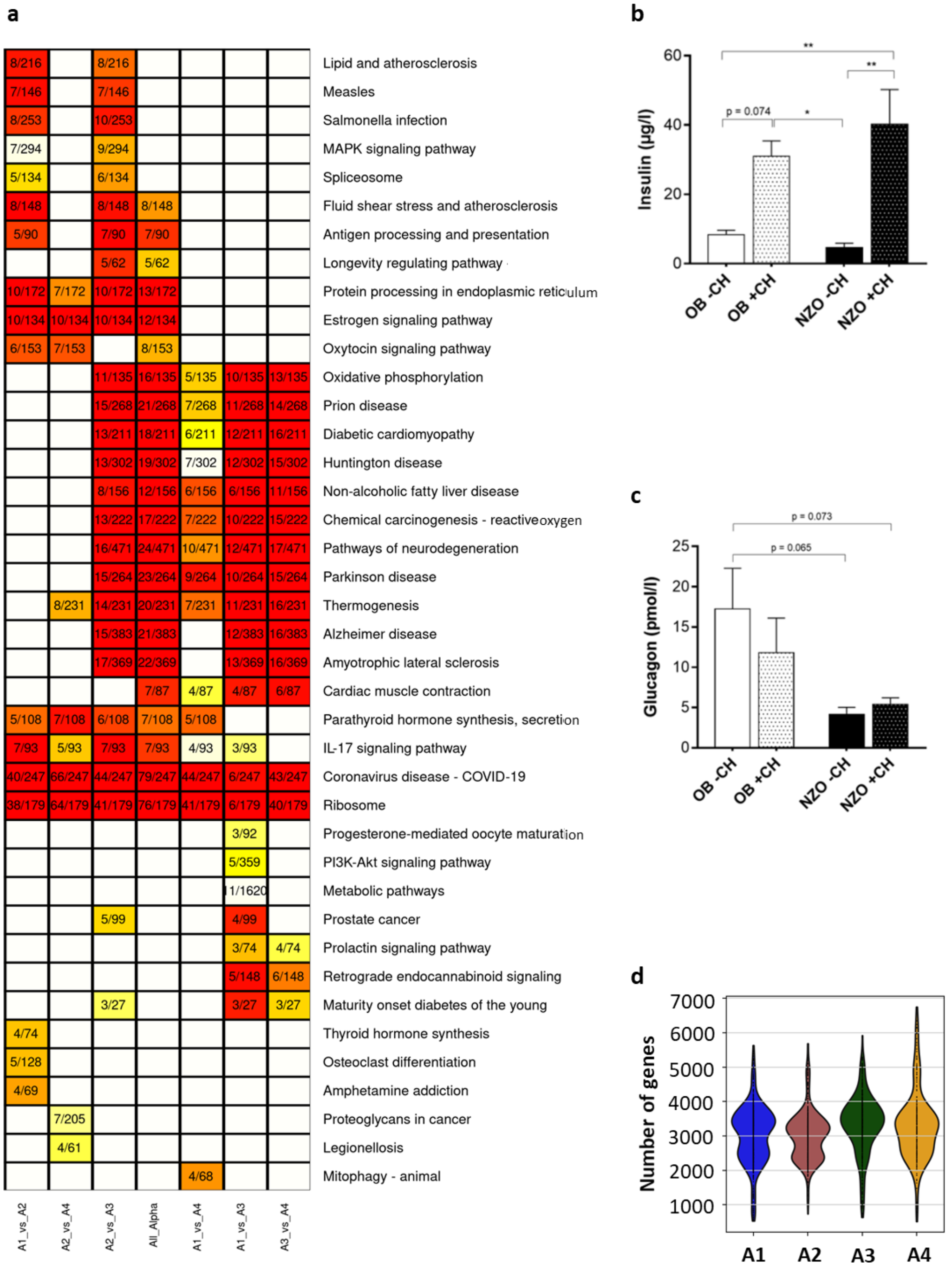

ESM Fig. 4. Characterisation of alpha-cell clusters by analysis of DEGs and hormone plasma levels under conditions with and without diabetogenic diet.

(**a**) Pathway enrichment analysis of DEGs between all clusters. First number depicts genes that are differentially expressed in the pathway, second number reflects number of genes within the pathway, and the colour codes for the p-value. Levels of plasma insulin (**b**) and glucagon (**c**) of OB and NZO mice fed with and without diabetogenic diet. Data are represented as mean ± SEM, and significance was determined using two-way ANOVA with Tukey's post hoc test (*p < 0.05, **p < 0.01). (**d**) Number of genes expressed in the different α-cell clusters.

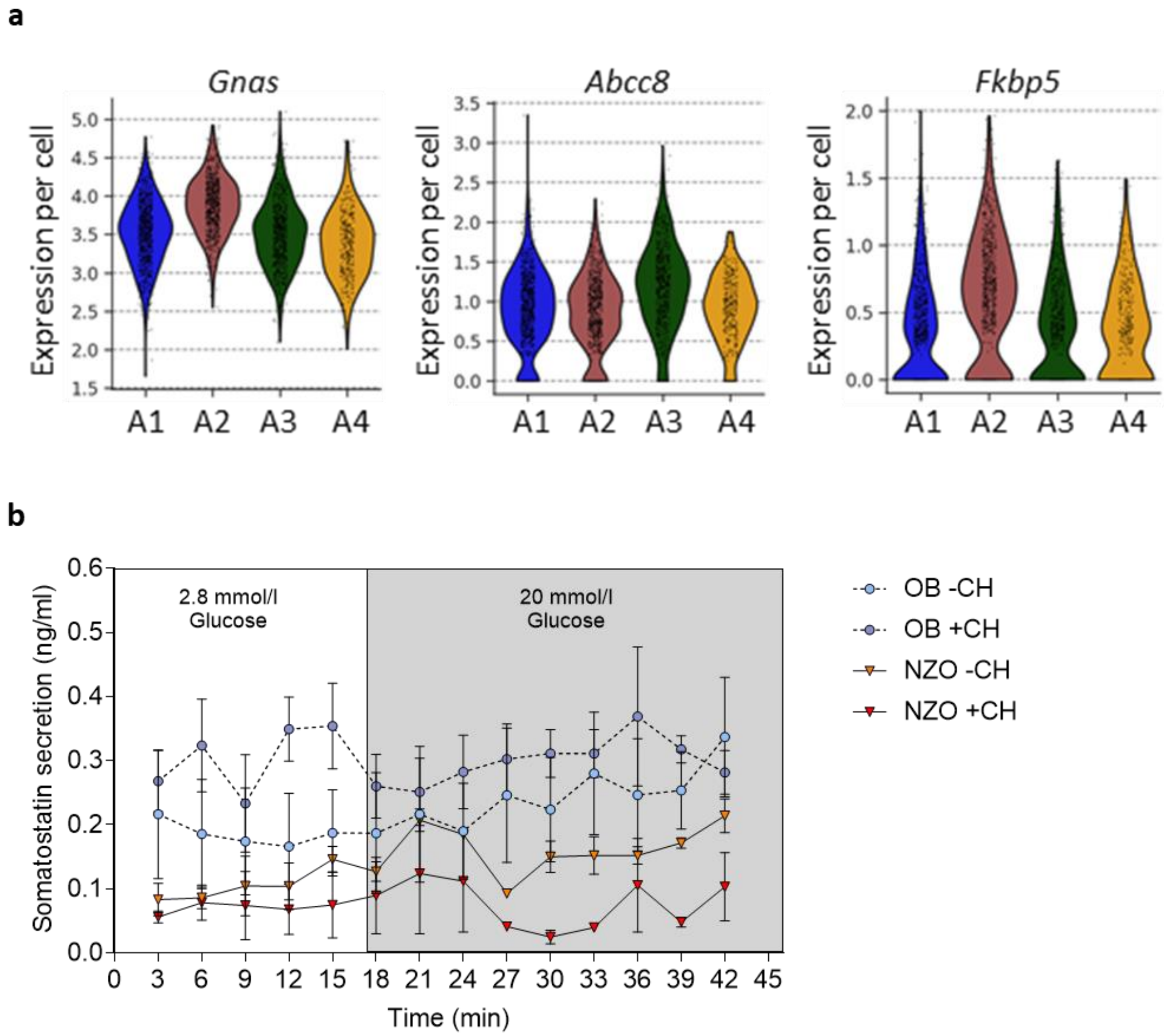

ESM Fig. 5. Expression of selected genes in alpha cell cluster and somatostatin secretion.

(**a**) Violin plots of selected genes. (**b**) Perifusion assay detecting somatostatin secretion of islets from OB and NZO mice under low and high glucose conditions (n = 2-3).

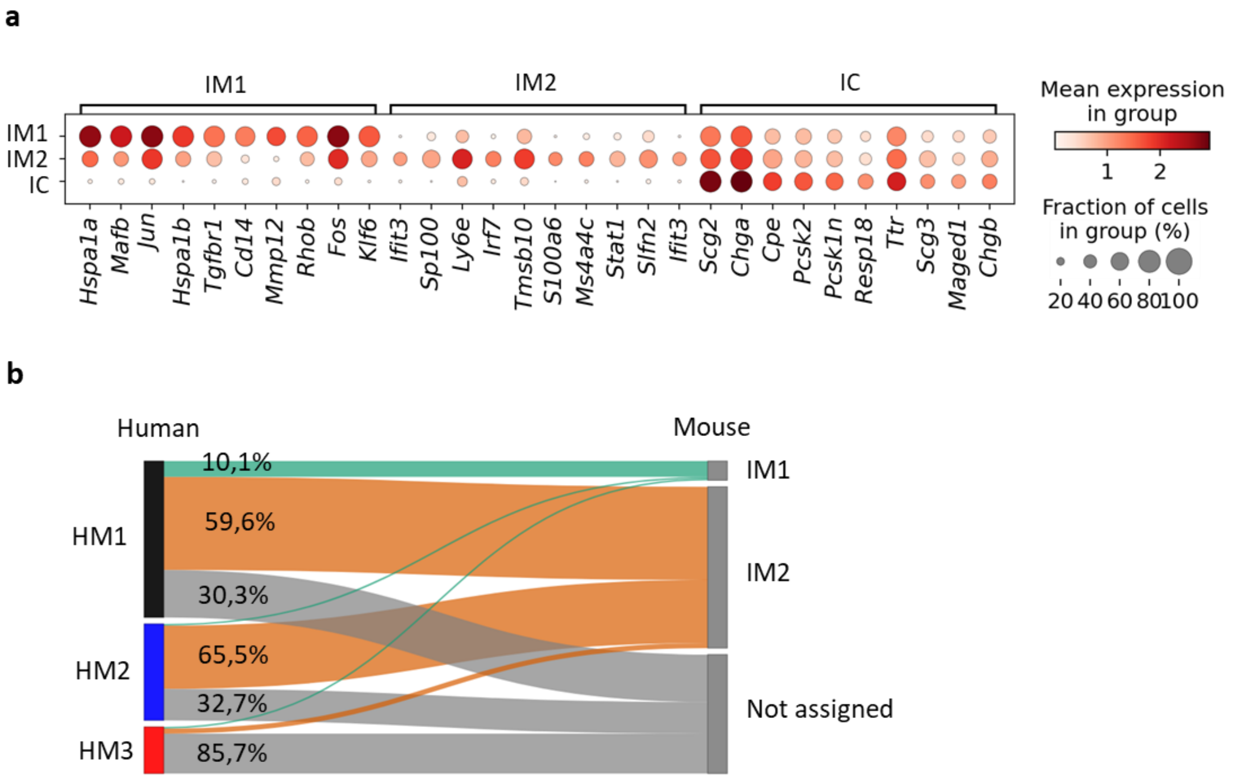

ESM Fig. 6. Macrophages in human and mouse islets.

(**a**) Dotplot depicting top 10 marker genes for macrophages (IM1/2) and immune cell (IC) clusters (**b**) Sankey-plot indicating the projection of human islets macrophages (HM) to mouse islets macrophages (IM).

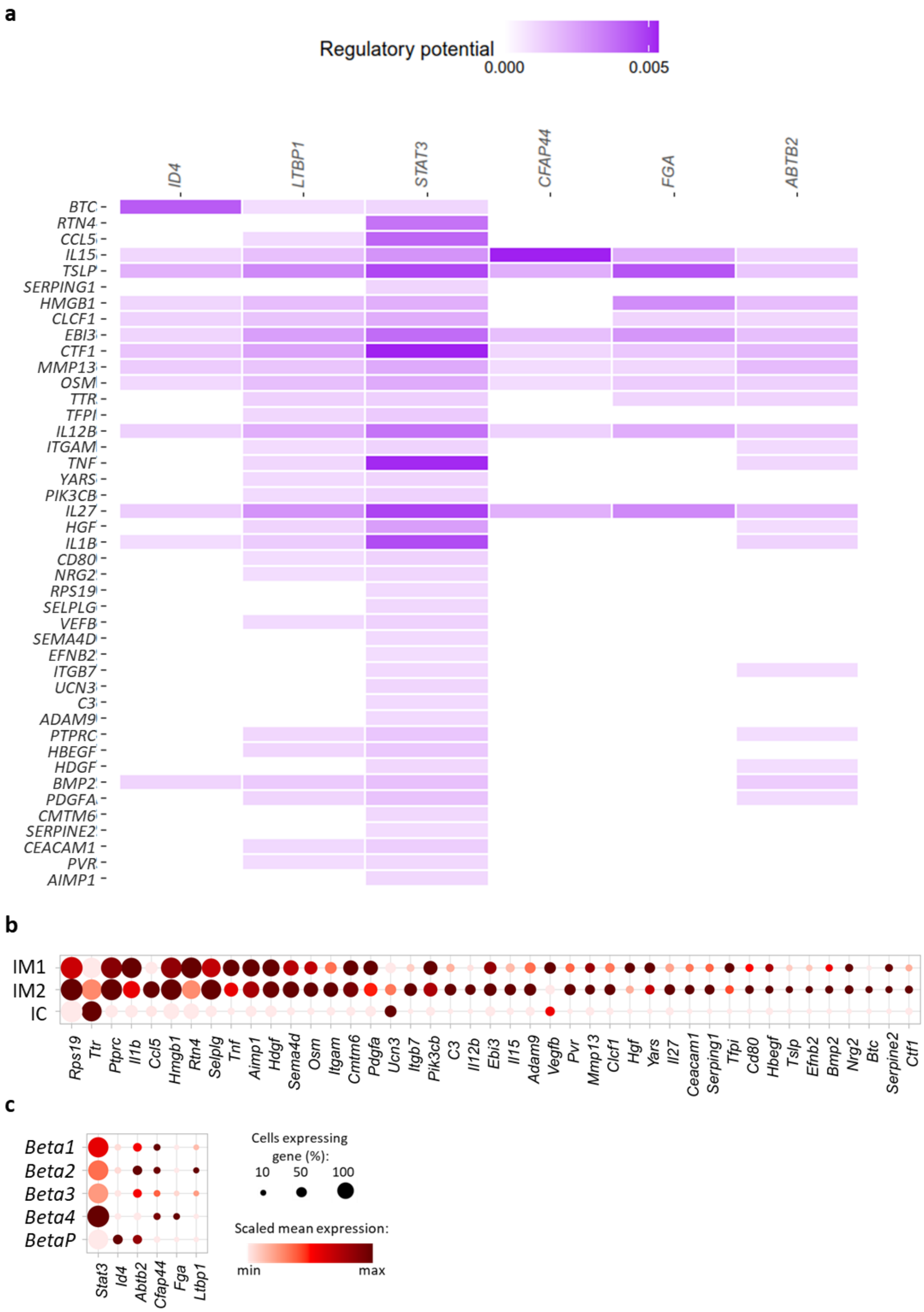

ESM Fig. 7. Heatmap displaying the regulatory potential of ligands on selected genes.

(**a**) Ligands are shown on the left side, while selected genes, including some encoding receptors, are shown on the top. Bright purple indicates stronger potential. This heatmap was created using NicheNet. Dotplots depicting the expression levels in the identified islet macrophages clusters of ligands (**b**) and putative targets (**c**) in the identified beta-cell populations from the NicheNet analysis.

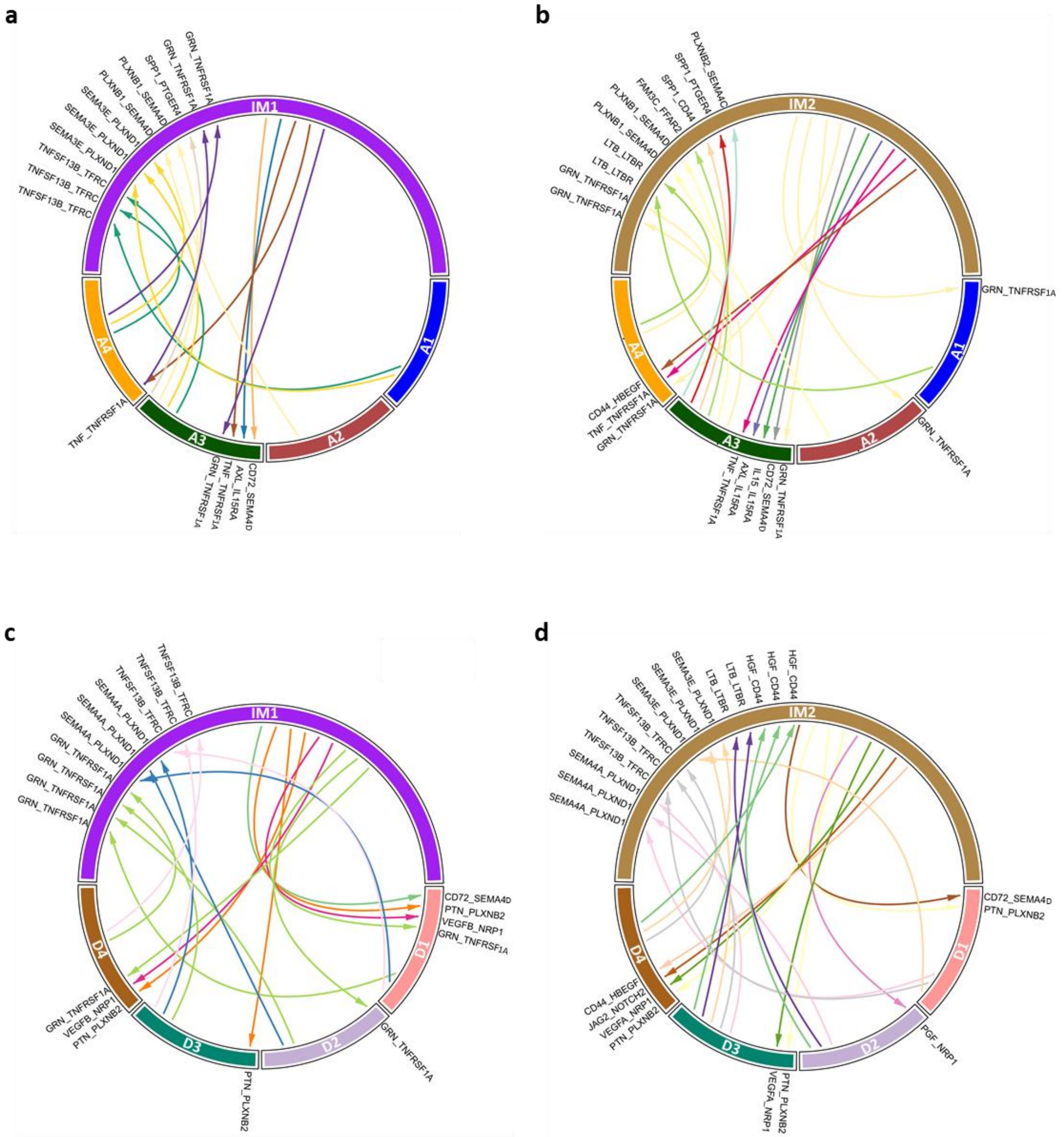

ESM Fig. 8. Cell-cell communication predicted via CellPhoneDB between alpha cells and delta cells with indicated islet macrophage clusters.

Cell-cell communication of clusters IM1 (**a**) and IM2 (**b**) with different alpha-cell clusters. Cell-cell communication of clusters IM1 (**c**) and IM2 (**d**) with different alpha-cell clusters.

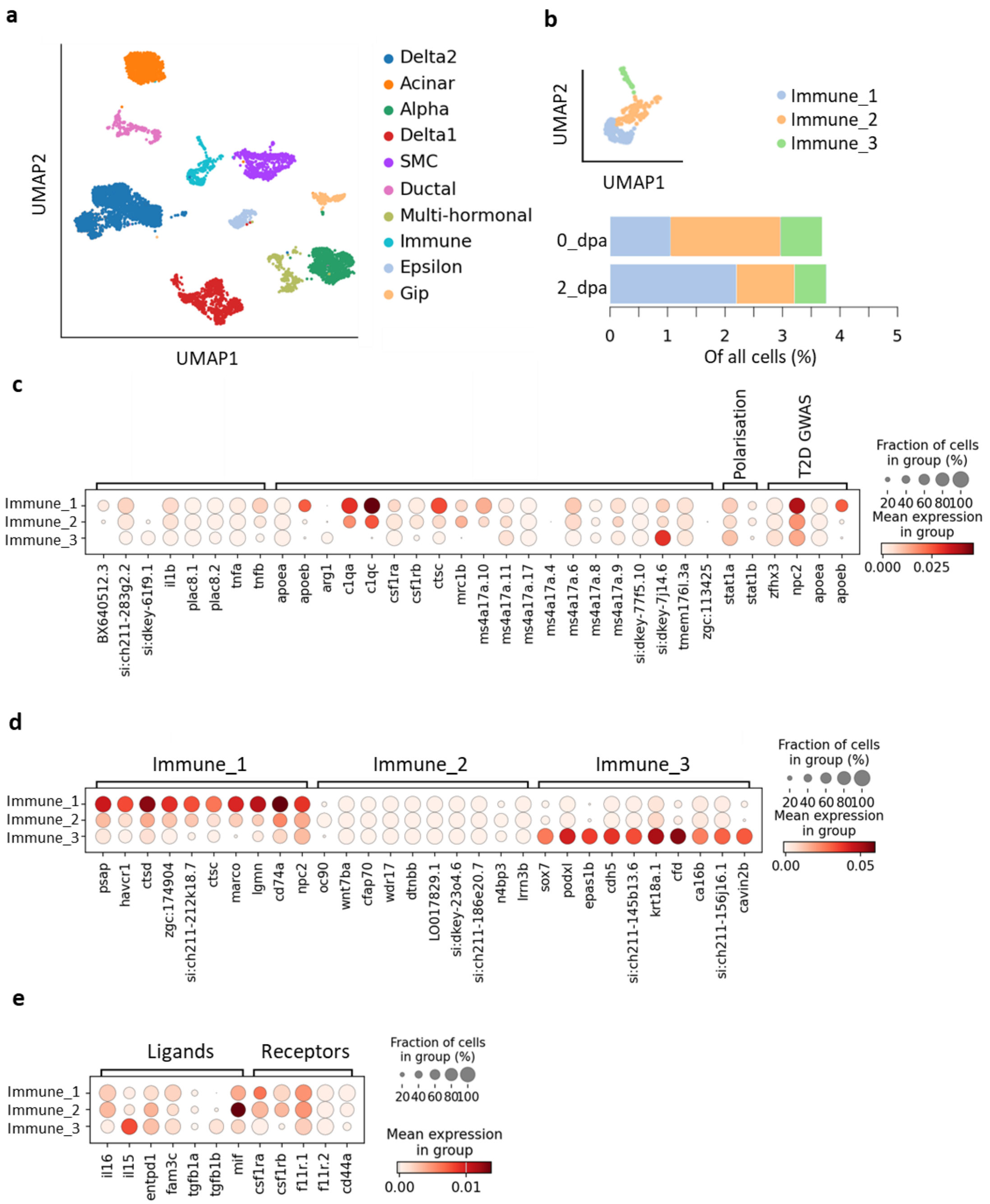

ESM Fig. 9. Identification of different macrophage clusters during beta-cell regeneration in zebrafish.

(**a**) UMAP plot of zebrafish islet cells 0 and 2 days after beta-cell ablation. (**b**) Re-clustering and quantification of macrophages in zebrafish 0 and 2 days after beta-cell ablation (dpa). (**c**) Expression levels of Mφ1 and Mφ2 marker genes, Stat1, and T2D risk genes in zebrafish macrophage clusters Immune_0-2. (**d**) Expression of top 10 marker genes to distinguish between different immune-cell clusters. (**e**) Expression of zebrafish ligands and receptors that were identified in the mouse dataset. UMAP: Uniform manifold approximation and projection

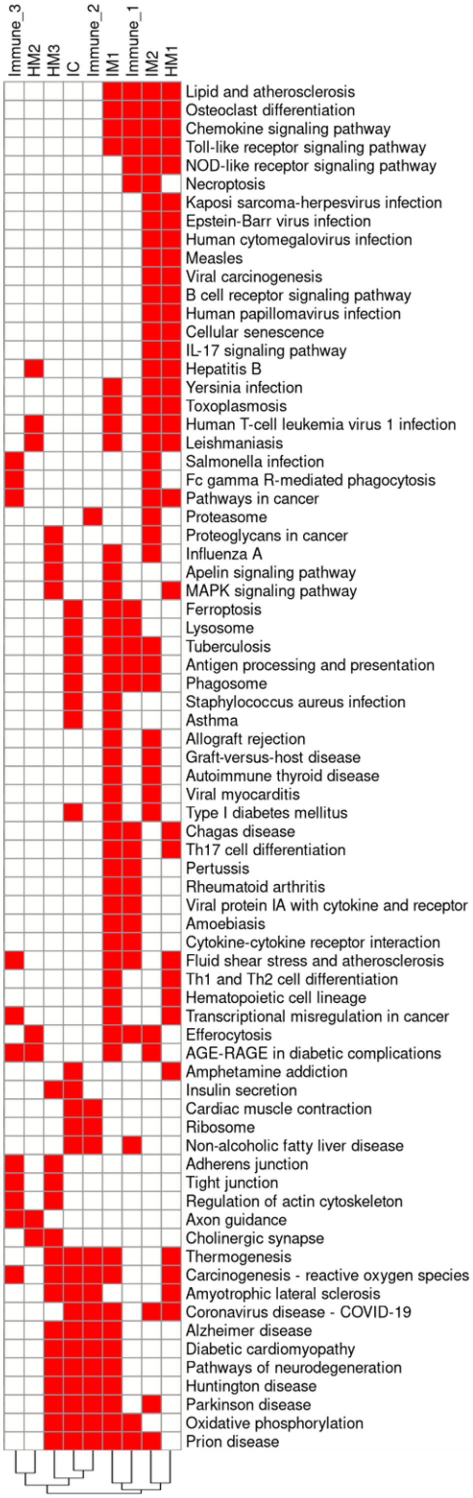

ESM Fig. 10. Pathway enrichment analysis of species-specific macrophage clusters.

Columns depict different islet macrophages from mouse (IM1, IM2 and IC), zebrafish (Immune_1, Immune_2 and Immune_3) and human (HM1, HM2 and HM3). Enrichment analysis was performed based on the top 250 marker genes.
